# N-acetylcysteine (NAC) ameliorates Epstein-Barr virus latent membrane protein 1 induced chronic inflammation

**DOI:** 10.1371/journal.pone.0189167

**Published:** 2017-12-11

**Authors:** Xiao Gao, Eirini-Maria Lampraki, Sarwah Al-Khalidi, Muhammad Asif Qureshi, Rhea Desai, Joanna Beatrice Wilson

**Affiliations:** College of Medical, Veterinary and Life Sciences, University of Glasgow, Glasgow, United Kingdom; University of Nebraska-Lincoln, UNITED STATES

## Abstract

Chronic inflammation results when the immune system responds to trauma, injury or infection and the response is not resolved. It can lead to tissue damage and dysfunction and in some cases predispose to cancer. Some viruses (including Epstein-Barr virus (EBV)) can induce inflammation, which may persist even after the infection has been controlled or cleared. The damage caused by inflammation, can itself act to perpetuate the inflammatory response. The latent membrane protein 1 (LMP1) of EBV is a pro-inflammatory factor and in the skin of transgenic mice causes a phenotype of hyperplasia with chronic inflammation of increasing severity, which can progress to pre-malignant and malignant lesions. LMP1 signalling leads to persistent deregulated expression of multiple proteins throughout the mouse life span, including TGFα S100A9 and chitinase-like proteins. Additionally, as the inflammation increases, numerous chemokines and cytokines are produced which promulgate the inflammation. Deposition of IgM, IgG, IgA and IgE and complement activation form part of this process and through genetic deletion of CD40, we show that this contributes to the more tissue-destructive aspects of the phenotype. Treatment of the mice with N-acetylcysteine (NAC), an antioxidant which feeds into the body’s natural redox regulatory system through glutathione synthesis, resulted in a significantly reduced leukocyte infiltrate in the inflamed tissue, amelioration of the pathological features and delay in the inflammatory signature measured by *in vivo* imaging. Reducing the degree of inflammation achieved through NAC treatment, had the knock on effect of reducing leukocyte recruitment to the inflamed site, thereby slowing the progression of the pathology. These data support the idea that NAC could be considered as a treatment to alleviate chronic inflammatory pathologies, including post-viral disease. Additionally, the model described can be used to effectively monitor and accurately measure therapies for chronic inflammation.

## Introduction

Human disease conditions displaying chronic inflammation frequently exhibit tissue degeneration, functional impairment and even permanent metabolic changes as a consequence [1]. Additionally, a link between chronic inflammation and increased cancer risk has been well documented [2, 3]. Activation of inflammatory responses involve the recruitment of leukocytes to a site, often mediated through factors released by mast cell degranulation (such as histamine) and by activation of macrophages which release proinflammatory cytokines (including tumour necrosis factor (TNF)-α). This process escalates with the sequential release of further inflammatory mediators (chemokines, cytokines and certain lipids) and the recruitment and activation of further inflammatory cells, which in turn release proinflammatory factors. Under normal circumstances, acute inflammation is self-limiting and though less well understood, the response abates after a few days when the infection or injury resolves. Persistent activation of inflammation or a failure to resolve the response, resulting in chronic inflammation, can lead to dysfunction, autoimmune disease and increased cancer risk.

During inflammation, phagocytes (including neutrophils, monocytes, macrophages and mast cells) produce oxidising enzymes (NADPH oxidase, inducible nitric oxide synthase (iNOS), xanthine oxidase and myeloperoxidase) leading to the generation of high levels of reactive oxygen and nitrogen species (ROS and RNS), which, in a feed forward loop, can promote further inflammation [4–7]. ROS and RNS act as signalling molecules at low concentration, but at the high concentrations released in a respiratory burst under inflammatory conditions, ROS and RNS can damage a wide range of biomolecules, including DNA, proteins and lipids [8]. Immune cells direct this action to eradicating infectious agents and infected cells, but under chronic inflammatory conditions, high level ROS and RNS can contribute to tissue damage [3].

There is wide interest in the potential of antioxidants as therapeutic agents to combat chronic inflammation and considerable controversy over the benefits of antioxidants verses oxidants in the prevention and treatment of cancer. N-acetylcysteine (NAC) is an antioxidant which is freely available over the counter. NAC is used clinically to treat a wide variety of medical issues and conditions [9], such as paracetamol overdose, acute respiratory distress syndrome, chemotherapy and heavy metal-induced toxicity; it’s also used as a mucolytic agent [10] and to ameliorate certain psychiatric disorders [11, 12]. Furthermore, NAC has been reported to have multiple other beneficial properties, such as enhancing bone regeneration, reducing tinnitus, reducing post surgical complications [13] and protective effects during mutagenic irradiation [14].

NAC is the acetylated precursor of cysteine, a semi-essential amino acid with a thiol side chain. Cysteine, can cross cell membranes (including the blood brain barrier) and is the rate-limiting molecule in the synthesis of the tripeptide glutathione (GSH; derived from glutamic acid, cysteine and glycine). GSH is a major endogenous antioxidant in the body, the thiol group serving as an electron donor in reducing reactions, during which GSH is oxidized to form glutathione disulphide (GSSG). Indeed GSH maintains vitamins C and E in their reduced (active) form. GSH has several essential functions in the body, as well as antioxidant activity (including scavenging ROS and RNS), it is involved in iron metabolism, it is used in biosynthesis, amino acid transport and enzyme activation. While NAC itself is a reducing agent, its rapid deacetylation *in vivo*, suggests that the effects of NAC *in vivo* largely reflect its action in maintaining GSH levels [9]. Consequently NAC (through GSH) can effect many biological processes, including mutagenesis and carcinogenesis, cell cycle, apoptosis, signal transduction and gene expression, immune modulation, the cytoskeleton, trafficking and neurotransmission, amongst others [9].

Clinical and animal studies have shown that NAC can reduce the acute inflammatory response to lung injury and sepsis and to myocardial injury, reducing tissue damage and improving impaired function [15–21]. However, in a long term study (24 weeks) NAC treatment of cystic fibrosis subjects revealed improved lung function in the treatment group, but no alterations in sputum inflammatory markers [22].

Inflammation following infection is a common complication, particularly after certain viral infections and occasionally bacterial. Epstein-Barr virus (EBV) is a notable contributor to inflammatory post-viral disease, including chronic fatigue syndrome following infectious mononucleosis [23], viral arthritis [24] and the life threatening condition of acute disseminated encephalomyelitis [25]. Indeed EBV is known to be a risk factor for the autoimmune disorders multiple sclerosis (MS) [26] and Systemic Lupus Erythematosus (SLE) [27], amongst others. In many cases there may be little evidence of active viral disease and the inflammation may reflect a footprint left by the response to the virus.

In this study, the impact of long term NAC treatment on chronic inflammation has been analysed, using a transgenic mouse model of chronic inflammation-associated carcinogenesis, induced by the latent membrane protein 1 (LMP1) of EBV [28]. LMP1-expressing cancers associated with EBV, including nasopharyngeal carcinoma (NPC) and the B-cell malignancy Hodgkin’s disease (HD), have an extensive leukocyte infiltrate in the tumours, to the degree that in the latter, the malignant cells make up only 1–2% of the tumour mass [29]. LMP1 displays multiple oncogenic properties and also acts as a pro-inflammatory factor [30]. Transgenic LMP1 expression leads to activation of several signalling pathways, including epidermal growth factor receptor (EGFR) pathways, constitutive activation of the JNK and NF-κB pathways and vascular endothelial growth factor (VEGF) upregulation [28, 31]. In the L2LMP1 mouse model, the transgene is expressed in epithelia; in the skin of the ears to the highest level [28]. As a consequence, phenotypic progression in this relatively hairless tissue can be readily monitored and we have categorized it into 5 pre-neoplastic stages. The inflammatory phenotype associated with these pathological stages has been previously described in detail [30]. At weaning (3 weeks old) the tissue shows increased vascularization (termed stage 1). This progresses within the next month to evident tissue thickening through hyperplasia and with the onset of an inflammatory infiltrate (stage 2). Over the next months the pathology worsens, showing increasingly severe hyperplasia, parakeratosis, ulcerative dermatitis, fibrovascular hyperplasia of the underlying dermis and necrosis (stages 3 through to 5). By 6 months of age, keratoacanthomas, papillomas and occasional carcinomas begin to form, both on the ears and other areas of skin [28]. The inflammatory infiltrate is mixed in this chronically inflamed tissue, involving activated CD8+ T cells, CD4+ T cells (including regulatory T cells), mast cells and neutrophils and a significant IgG deposition occurs in the dermis [30]. Multiple pro-inflammatory cytokines and chemokines (including IL-1β, CCL3, CCL5, CXCL1, CXCL2 and CXCL5) become upregulated as the pathology progresses, along with several other leukocyte attractants and trafficking factors (including CCL19, CCL20, CCL27, CXCL10, CXCL13, XCL1 and L-selectin). T-helper cell specific cytokines also become upregulted; at early phenotypic stages a mixture of Th1 (IFNγ, IL-2 and CXCL16), Th2 (IL10 and IL13) and Th17 (IL17) responses co-exist, while at later stages (stage 4/5) a more destructive Th1 response dominates, consistent with the necrotic phenotype [30].

The importance of inflammatory pathways in EBV associated carcinogenesis has recently been highlighted in an extensive exome sequencing study of NPC samples, both in terms of somatic carcinogenic mutations and germ-line susceptibility loci [32, 33]. Additionally, the noted association of EBV infection with multiple post viral inflammatory and auto-immune conditions renders this a particularly focus for therapeutic approaches. As such, agents that act to limit chronic inflammation may be of benefit both to non-malignant chronic inflammatory disorders as well as in the treatment of some inflammation-associated malignancies.

Here, we report that treatment of L2LMP1 mice with NAC depresses and delays the chronic inflammatory phenotype and we have quantified this by *in vivo* imaging.

## Materials and methods

### Mice

The L2LMP1 transgenic mouse line 117 (LMP1 of CAO EBV strain, aka LMP1^CAO^, linked to L2 expression control sequences), in the FVB mouse strain was used in these studies, with transgene negative siblings used as controls (NSC), as previously described [28]. CD40 null mice (in a C57Bl/6 background) [34] were crossbred with L2LMP1 mice, back-crossed 4 times to the FVB strain and then inter-crossed to establish LMP1/CD40KO (93.75% FVB). Phenotype scoring was conducted on a weekly basis and compared to CD40 wild type mice of the same strain mix background. Ear pinnae thickness was measured with calipers. Mice were monitored at least twice weekly to assess health and well-being. Mice were removed from study using an approved schedule 1 method. Tissue samples were frozen in liquid N_2_ and stored at -70°C for sample extraction. For NAC treatment, NAC was supplied in the drinking water at 10g/dm^3^ and the water was replaced every 3 days.

### Ethics statement

All animal work was conducted, all protocols approved and this study was approved, under UK Home Office license and according to institutional, national (Animal Welfare and Ethical Review Body, AWERB) and international guidelines: the UK’s Animals (Scientific Procedures) Act 1986 as amended by Animals (Scientific Procedures) Act 1986 Amendment Regulations 2012. This law is derived from the EU Directive on the Protection of Animals used for Scientific Purposes (Directive 86/609/EEC as updated by Directive 2010/63/EU).

### Isolation of leukocytes from ear tissue

Cells were isolated from ear tissue, essentially as described [30]. Briefly, the collected tissue was finely minced in 2ml PBS/ear, then 10mg/ml collagenase II, 10mg/ml collagenase IV, 0.5mg/ml DNaseI, CaCl_2_ to 3mM, were added and incubated at 37°C for 30 mins (shaking). Then dispase-II was added to 5mg/ml and incubated 15 mins, 37°C (shaking). To the cell samples (per 1 or pair of ears), two volumes of PBS + 10%FCS was added and then passed through a 40μm filter to remove clumps (with a follow through filter wash of 5ml PBS). Collected cells were washed 2x with 10ml PBS (centrifugation 194xg, 10 mins). Then, either the whole sample or 10^6^ cells per sample was retained for flow cytometry.

### Leukocyte transfer in vivo

Isolated and washed cells from the ears of 2 mice (approximately 10^7^ cells after removal of an aliquot for flow cytometry) were re-centrifuged and resuspended in 10ml complete medium (RPMI, 20% FCS, 1%glutamine, 1%penicillin/streptomycin (Sigma)) and incubated 37°C overnight. Suspension cells were collected, counted, washed in PBS and resuspended in 300μl complete medium and stained with cell proliferation dye (CPD eBioscience) at 80μM, for 5 mins at RT. Cells were washed 2x with 5ml PBS and resuspended at 10^6^cells/150μl PBS and kept on ice for IV injection into the tail vein.

### In vivo imaging system (IVIS)

For imaging CPD stained cells *in vivo*, mice were inhalation anaesthetised with 4% isofluorane and 0.5L/min oxygen. Epi-fluorescence imaging was conducted using λ 640nm excitation and 680nm emission, first at low magnification to view the passage of cells from the tail and subsequently at higher magnification to examine ears. Images were acquired and analysed using LivingImage v4.3.1 (PerkinElmer), binning setting of 4, exposure time 1 second. Fluorescent images were captured from 1 to 48 hours post injection (hpi) and regions of interest (ROI) were drawn around the ears to obtain the average radiance efficiency (ARE) in ([photons/s/cm^2^/sr]/[μW/cm^2^]).

For inflammation imaging *in vivo*, mice were injected IV with 0.3 to 2nmol ProSense 680 (p680, PerkinElmer) in 200μl PBS. Mice were anaesthetised at time points, from 1 to 48 hpi and fluorescent images captured using λ 675nm excitation and 720nm emission. Images were analysed and radiance from ROI quantified.

### Western blotting

Proteins were extracted in RIPA buffer and were separated (30–100μg per track) by SDS-PAGE (7.5%, 10% or 15%); blotting and washing were performed as previously described [30]. The blots were incubated in 5% non-fat milk PBS 0.1% (v/v) Tween 20 with the appropriate antisera. Antibodies (with dilutions) used were directed to: LMP1 (IG6) 1:100, TGFα (Santacruz) 1:1000, Chil3/4(YM1/2) (R&D systems) 1:1000, SOD1 (Santacruz) 1:200, Erk1/2 (Cell Signaling) 1:1000, phosphor-Erk1/2 (Cell Signaling) 1:1000, Stat3 (Cell Signaling) 1:1000, S100A9 (R&D Systems) 1:1000, EGFR (Cell Signaling) 1:1000, IκBα (Cell Signaling) 1:1000, phosphoIκBα (Cell Signaling) 1:1000, GAPDH (Santacruz) 1:1000, actin (Santacruz) 1:1000, IgE (Abcam) 1:1000, followed by the appropriate 1:4000 goat anti-mouse, anti-rabbit, anti-rat, or donkey anti-goat IgG HRP-conjugates (Santacruz). Mouse IgM, IgA and IgG were detected directly using antibody HRP-conjugates (Santacruz or Southern Biotech.) 1:4000. Detection was performed by enhanced chemiluminescence (liteAblot kit, Euroclone) and bands on blot images quantified using imageJ.

### Flow cytometry

To cells in 300μl to 700μl PBS, TruStain (BioLegend) was added at 1μg/100μl for 10 mins on ice. Cell aliquots (10^5^ to 10^6^) were made up to 200μl with PBS and incubated with fluorescent marker conjugated antibodies directed against: CD45-FITC or CD45-APC (BioLegend) at 37°C, 25 mins. For ROS detection, 2μl of DCFH-DA was added to each sample at the same time as anti-CD45 and samples were incubated at 37°C, 15 mins. Samples were washed with 1ml PBS (centrifugation 400g, 5 mins) and either washed again and resuspended in 500μl PBS, 1% FCS for FACS or fixed in 100μl 2% buffered paraformaldehyde, 15 mins dark. Fixed cells were centrifuged and resuspended in 100μl PBS and stored in the dark, overnight, 4°C. Before analysis, fixed cells were pelleted and resuspended in 500μl PBS, 1% FCS. Samples were sorted using FACSCalibur or FACSAria (BD Biosciences) and data acquired using FlowJo and analysed using Kaluza 1.2 (Beckman-Coulter). Statistical analyses were carried out using GraphPad Prism 5, using paired T-tests.

## Results

### Immunoglobulins of all isotype groups are detected in the inflamed tissue

**Fig 1:**
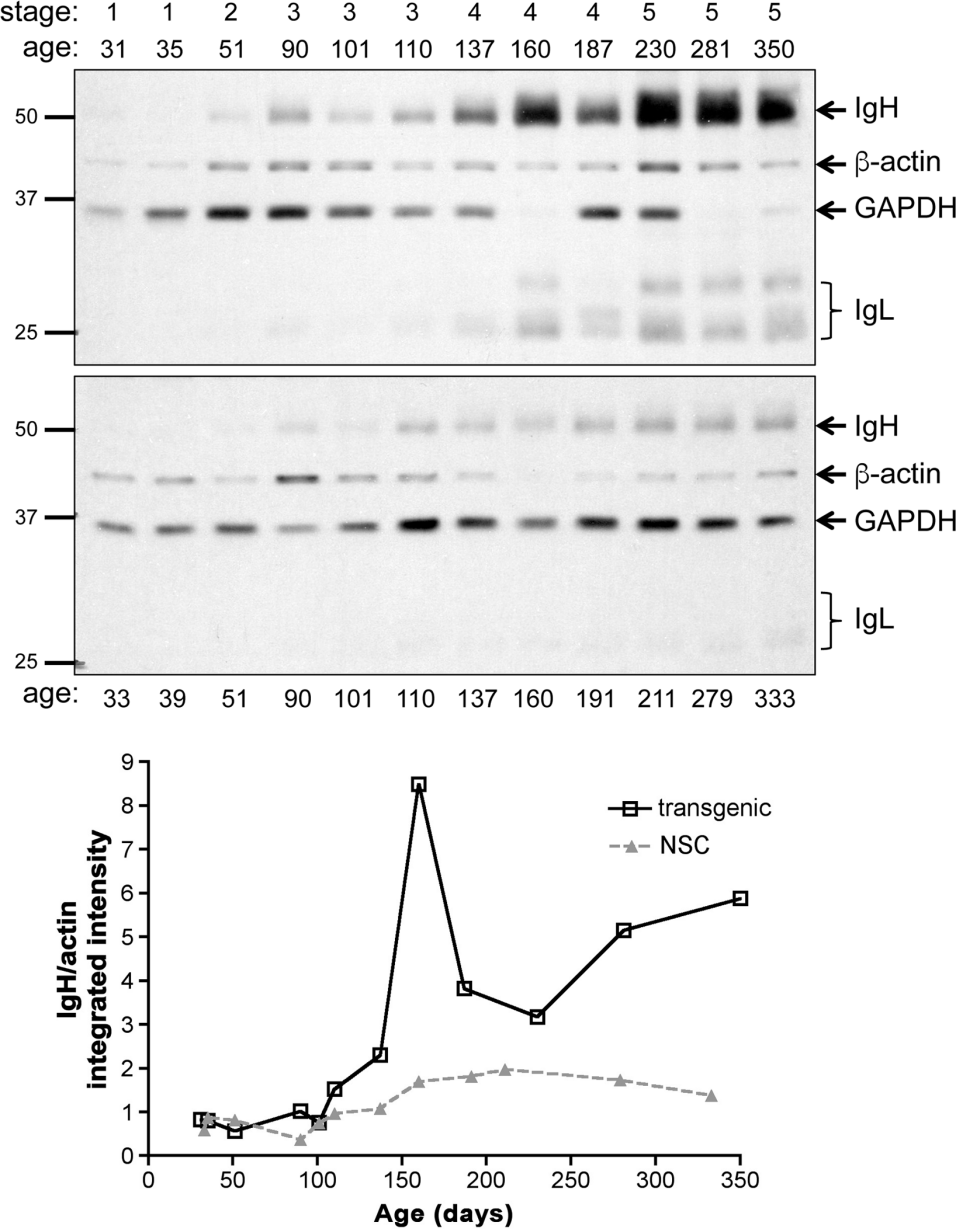
IgG deposition in the transgenic ear tissue increases with phenotypic stage and age. Protein (50μg/track) was extracted from L2LMP1 transgenic ears (stages 1 to 5, upper blot as indicated) and control (NSC lower blot) of mice of increasing age and western blotted (age in days, as indicated above or below (respectively)). The blots were probed with antibodies to actin (42kD) and GAPDH (37kD), using anti-mouse IgG as secondary, also detecting tissue immunoglobulin-G: IgH and IgL, as indicated. Protein size markers in kD are indicated to the left of each panel. Below: the normalised values (IgH/actin) are shown graphically for transgenic (L2LMP1) and NSC samples, plotted against age.

LMP1 acts as a proliferative and pro-inflammatory factor in the skin of L2LMP1 transgenic mice, particularly evident where the transgene expression is highest, in the ear skin. The phenotype has been categorised by visible parameters into stage categories (described above) [28], supported by ear thickness measurements. At stage 2, prior to the appearance of haemorrhagic or necrotic foci, the ear pinnae were significantly (p<0.0001) thickened compared to transgene negative sibling controls (NSC), by approximately 3 fold, from a mean of 0.21mm (SD +/-0.05) in NSC mice of all ages to a mean of 0.6mm (SD+/- 0.19) (p = 0.001). The pinna thickness increases further with progressive stage. The advancing phenotype can also be evidenced by examination of immunoglobulin (Ig) deposition in the dermis (previously shown by western blotting and immunohistochemistry) [30]. Tissues categorised from stage 3 to stage 5 show consistently more IgG deposition in the tissues compared to controls (Fig 1).

**Fig 2:**
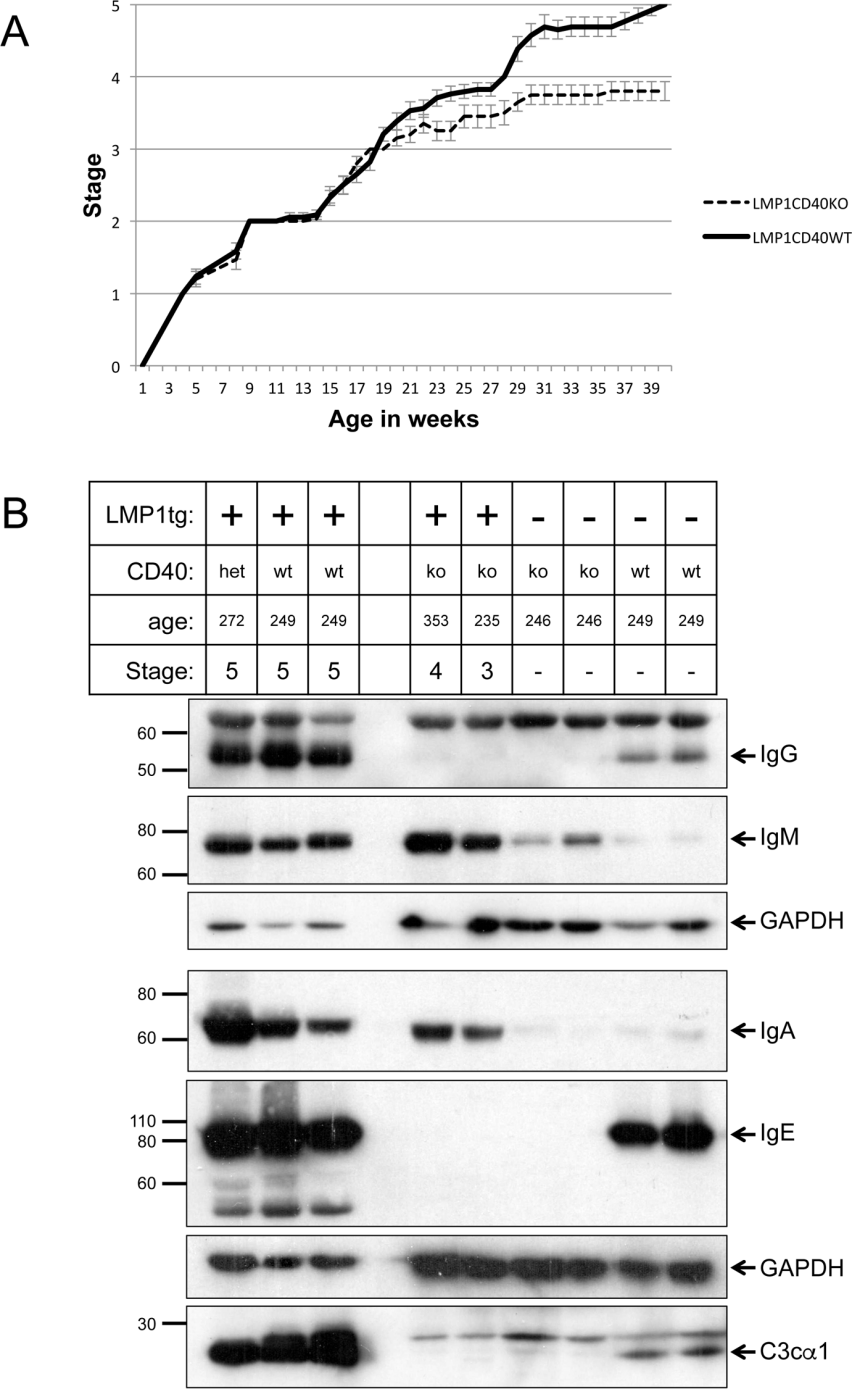
IgG, IgM, IgA, IgE and complement C3c levels are increased in the transgenic tissue. (A) The phenotypic stage was monitored in a cohort of LMP1 transgenic mice in a wild type background (LMP1CD40WT: n = 17) compared to LMP1 transgenic mice in a CD40 null background (LMP1CD40KO: n = 15) and plotted over time; error bars show SEM. (B) Ear tissue protein samples from mice of the following genotypes: L2LMP1 transgene (LMP1tg) positive (+) or negative (-), in either a CD40 null background (ko), heterozygous (het) or wild type for CD40 (wt) were western blotted. Age in days and phenotypic stage are indicated. The blots were probed with antibodies to mouse IgG, IgM, IgA, IgE and complement C3 and re-probed with antibody to GAPDH (37kD). Protein size markers in kD are indicated to the left of each panel. Note: IgG, IgE and C3c are not produced in CD40 null mice, however, IgM and IgA are induced in the LMP1 transgenic tissues.

We have previously observed that CD40 is strongly upregulated in the transgenic inflamed skin [30] and it is also expressed in NPC tumour samples [35]. CD40 has broad functions in immune and inflammatory responses, but in particular, it is a potent activator of B-cells and is essential for immunoglobulin class switching to IgG and IgE, memory B-cell development and germinal centre formation [34]. Additionally it has been observed that LMP1 can substitute for some functions of CD40 [36]. To explore the role of CD40 and B-cell activation in the transgenic LMP1 induced inflammation, L2LMP1 mice were cross-bred into a CD40 null background. There was no observable impact upon the early hyperplastic and inflamed stages (stages 2 and 3) of the LMP1-induced phenotype in the CD40 null background, however the mice showed a significant reduction in the ulcerative and necrotic lesions evident in the highly inflamed tissues at stage 4, with none advancing to stage 5 (Fig 2A). In a CD40-wild type background, IgG, IgM, IgA, IgE and the cleaved complement protein C3c were all induced in the L2LMP1 inflamed skin compared to NSC (Fig 2B). Western blotting confirmed the absence of IgG and IgE, as well as complement C3c, in the CD40 null skin tissue, however, the LMP1 transgenic samples nevertheless showed an increase in IgM and IgA levels compared to NSC, in both a CD40-wild type and CD40 null background. These data show that CD40 action (including IgG and IgE production and complement system activation), contributes to the tissue degenerative aspect of the inflammatory phenotype seen in older mice, without apparent contribution to the early, proliferative features of the phenotype.

### NAC treatment delays the progression of the inflammatory phenotype

**Fig 3:**
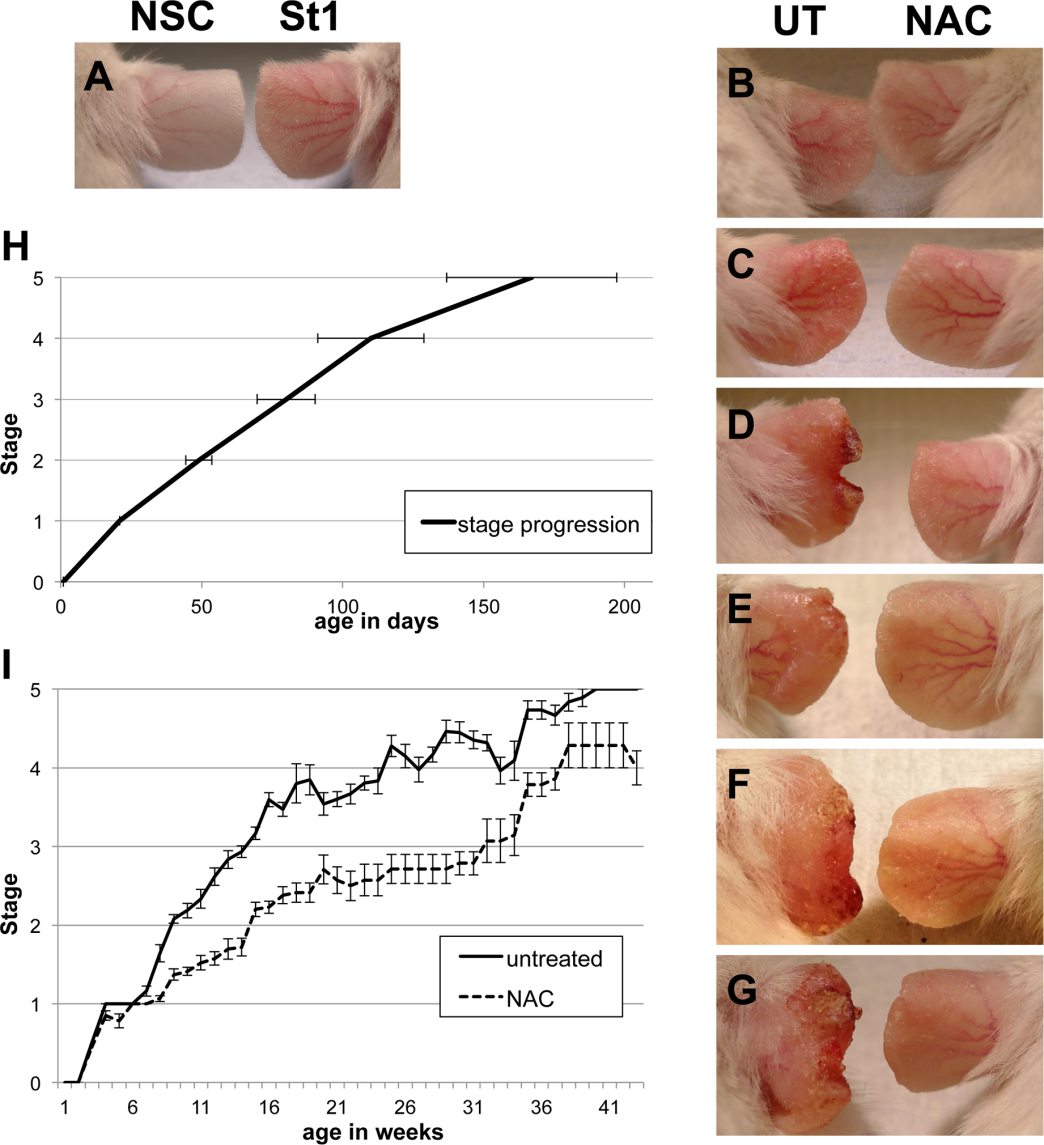
NAC treatment delays phenotypic progression. A to G photographs of ear skin phenotype: A: The phenotype of a transgenic mouse (right) at 42 days old, referred to as stage 1 (St1), compared to NSC (left). (B-G): The phenotypic effect of systemic treatment of transgenic mice with NAC (right) from 38 days old, compared to untreated (left: UT) at progressive ages and phenotypic stages. Age and stage details: B: 42 days old: left: untreated stage 1, right: 4 days NAC. C: 60 days old: left: untreated stage 2, right: 22 days NAC. D: 84 days old: left: untreated stage 3, right: 46 days NAC. E: 100 days old: left: untreated stage 3, right: 56 days NAC. F: 140 days old: left: untreated stage 4, right: 96 days NAC. G: 160 days old: left: untreated stage 5, right: 116 days NAC treatment. (H) The mean age of progression to the next phenotypic stage is shown graphically for untreated transgenic mice (n = 57, error bars show SD). (I) The average phenotypic stage with age is plotted for a cohort of transgenic mice treated with NAC (total n = 34, with at least n = 7 for each time point) compared to untreated (total n = 188, with at least n = 10 for each time point). Stage progression is significantly different between the two groups (P<0.0001) from 7 weeks of age onwards. Error bars show SE. Note: drops in the curve for untreated mice do not reflect phenotype reversal but are due to removal of mice from study.

In order to explore if the feed-forward loop between oxidative stress and chronic inflammation could be disrupted, the effect of long-term NAC treatment was examined. Under acidic conditions (such as gastric fluid), NAC can cross cell membranes, but once in the systemic circulation, under neutral conditions, negatively charged NAC cannot cross intact, unimpaired membranes, however, with deacetylation, cysteine can [9]. The half-life of NAC in the body, administered by intravenous injection, is approximately 5.6 hours, with 30% cleared by renal excretion [9]. Therefore, in order to provide a continuous NAC regimen, transgenic and NSC mice were treated with NAC in the water supply, either from birth or starting at approximately 1 month of age (30–40 days old). Supplying NAC-water postpartum resulted in a reduced pup weight at weaning for transgenic, but not wild type mice, while treatment from 1 month of age caused no weight loss. Subsequently, all treatments were initiated in mice at 1 month old (at which point transgenic mice show a stage 1 phenotype) and treatment was generally continued throughout life. The phenotypic progression was monitored in NAC treated and untreated L2LMP1 transgenic mice. Mice treated with NAC showed significantly slower phenotypic progression (p<0.0001 from 7 weeks of age onwards), with a prolonged period transiting stages 1 to 3 (Fig 3). In addition, some NAC treated mice showed a temporary phenotype reversal, reverting from stage 2 to a stage 1 or 1/2 intermediary state, before slowly progressing on again to stage 2 some weeks later (S1 Fig). However, given time, the phenotype could reach stages 4 and 5 in some NAC treated mice. It was observed that the earlier the age the treatment was started, the more pronounced the retardation of phenotype progression (S1 Fig).

### In vivo quantification of the inflammatory phenotype

In order to develop a quantitative, objective measure of the inflammatory phenotype, the utility of *in vivo* imaging using a protease activatable fluorescent agent (p680) was assessed. Optically silent in its unactivated state, p680 becomes highly fluorescent when cleaved by lysosomal proteases (primarily cathepsin B, L, S and plasmin), which are frequently induced during inflammation [37, 38]. Utilising p680 with IVIS in L2LMP1 transgenic and NSC mice was optimised (S1 File), and subsequently, using between 0.66 and 1nmole p680/mouse was selected, with the *in vivo* fluorescent signal from the ears peaking between 24 and 36 hpi.

To determine if p680 with IVIS can be used to monitor the progressive phenotype and its retardation with NAC treatment in the L2LMP1 mice, transgenic and NSC mice at increasing ages and phenotypic stage were imaged. Mice in approximate age groups of 57, 80, 100 and 130 days old were imaged, the transgenic, untreated mice displaying a phenotype ranging from stage 1 to advanced stage 4 in these groups (Figures A to D and Table A in S2 File). Mice were imaged at intervals between 3 and 36 hpi, taking the radiant efficiency of the region of interest (ROI) (Figure E in S2 File).

**Fig 4:**
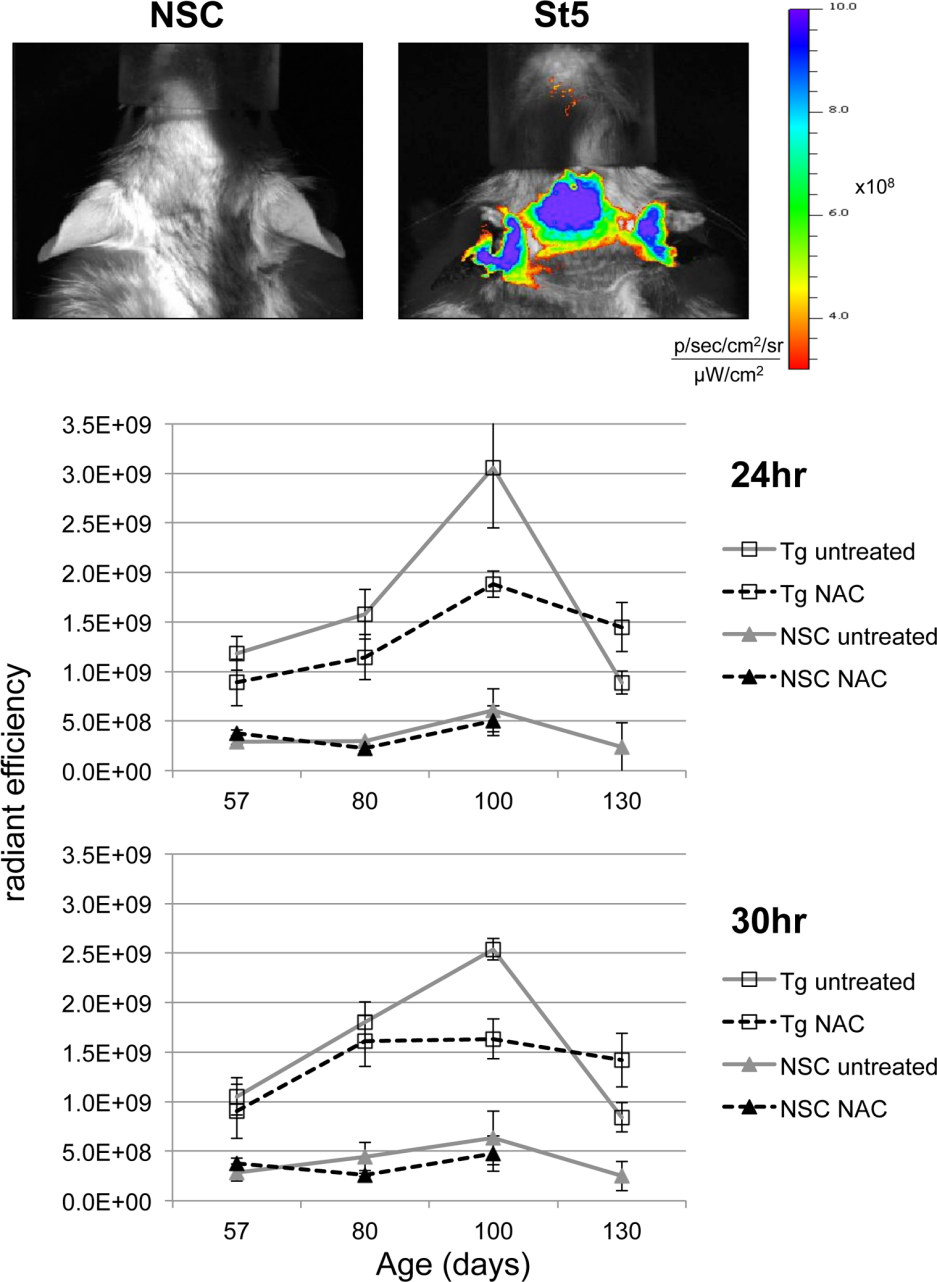
NAC treatment reduces the quantifiable levels of chronic inflammation in the L2LMP1 transgenic mice. Top: typical epifluorescent image taken 24 hours after IV injection of p680 into a transgenic L2LMP1 mouse at phenotypic stage 5 (St5) and negative sibling control (NSC); colour scale shows radiant efficiency range displayed ((photons/sec/cm^2^/sr)/(μW/cm^2^)). Below: graphs depicting the mean (with SD error bars) radiant efficiency (Y axis) observed in mice in increasing age groups (X axis) imaged at 24 and 30 hours (as indicated) post injection of 1nmol/mouse p680. Four groups were examined, transgenic (Tg) and NSC mice, either untreated or treated with NAC-water. N = 2 to N = 8 for each age and group (as detailed in Table A in S2 File).

The radiant efficiency measured from NSC mice showed little variation with age, averaging 3.1x10^8^ at 24 hpi and 3.3x10^8^ at 30hpi. L2LMP1 transgenic mice all showed significantly higher readings at all ages and stages, at 24 and 30 hpi averaging 1.5x10^9^ and 1.4x10^9^ (respectively), an order of magnitude higher than NSC (p<0.0001) (Fig 4 and Figures F and G in S2 File). The radiant efficiency from transgenic mice varied with age and stage, showing a 2 to 3 fold increase in mice aged 100 days old (stage 3) compared to 57 days old (stage 1). In older mice (130 days), at phenotype stages 4 and 5, the radiant efficiency decreased to levels similar to the young transgenic mice at stage 1. Although the phenotype becomes visibly more severe at stages 4 and 5, the overall reduction in fluorescent signal (compared to stage 3 phenotype) could be explained by the degeneration of the tissue, the ears become smaller, with the necrotic regions obscuring the fluorescent signal coming from live tissue underneath (see Fig 3).

Treatment of the L2LMP1 transgenic mice with NAC resulted in a significantly reduced fluorescent signal in mice up to 100 days old (P = 0.03), consistent with a retardation of the observed phenotype (Fig 2). However, by 130 days of age, the signal from untreated mice declined (with increasing necrosis), while the signal from NAC treated mice (largely free of necrosis at this point) showed a slower decline and the difference is significant (P = 0.0003). The data reveal that the degree of inflammation (as measured by reagent p680) increases more slowly in transgenic mice treated with NAC, reaching a lower peak before the onset of necrosis, compared to untreated mice. This is consistent with the phenotypic observations and provides a quantitative measure of the effect of treatment.

### NAC reduces the leukocyte load in the inflamed tissue

**Fig 5:**
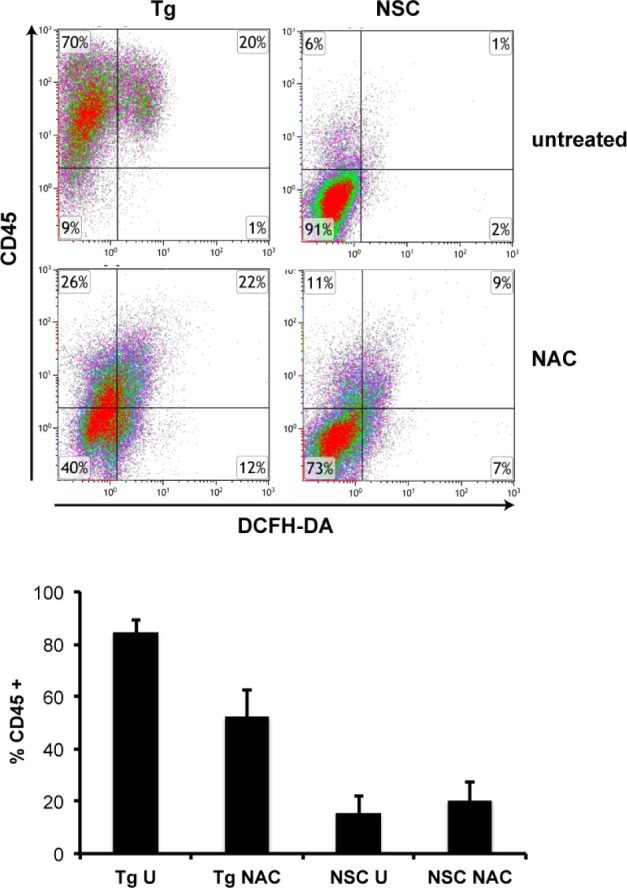
NAC treatment reduces the number of leukocytes in the inflamed tissue and their oxidative status. Cell suspensions were generated from ear tissue from mice aged approximately 105 days old. Samples were taken from L2LMP1 transgenic mice (Tg) treated with NAC (n = 6) or untreated (U: n = 7) and NSC treated with NAC (n = 3) or untreated (n = 3) (as indicated) and analysed by flow cytometry. Live cells were gated (staining negative for 7AAD) and the proportion of leukocytes (CD45+) assayed and examined for evidence of intracellular ROS through DCFH-DA. Top panels show representative dot plots obtained for each category. The percentage of CD45+ cells, as quantified using the quadrant values (top right and top left quadrants combined) is graphed below. The difference between Tg U and Tg NAC and the difference between Tg U and NSC U is statistically significant (P<0.0001).

The leukocyte infiltrate in the inflamed tissue has been characterised previously [30]. Treatment of the L2LMP1 mice with NAC resulted in a visible and quantifiable delay in the progressive inflammatory phenotype. In order to assess both the impact on leukocyte load and levels of intracellular reactive oxygen species, samples were examined by flow cytometry. Cells were isolated from ear tissue from transgenic and NSC mice, either treated with NAC or untreated, at 105 days old. Dead cells were excluded by 7AAD staining and live cells were stained for CD45 (a pan leukocyte marker) and intracellular 2’,7’-dichlorofluorescein-diacetate (DCFH-DA), a cell permeable agent which reacts with H_2_O_2_ (and other ROS) to fluoresce. The inflamed transgenic tissues carry a high leukocyte load compared to NSC and a substantial proportion of these cells have high intracellular ROS (Fig 5). On average, 85.6% of the isolated cells from transgenic ear samples were positive for CD45, showing a discrete leukocyte population and a distinct sub-population of leukocytes (average 20% of the total leukocytes) also staining for intracellular ROS (DCFH-DA), possibly undergoing a respiratory burst. By contrast, significantly fewer cells were CD45+ in NSC mouse samples (both treated with NAC or untreated). In addition, NSC samples did not show a discrete proportion of cells staining for intracellular ROS, with quadrant values significantly lower than transgenic samples (S2 Fig). Treatment of the transgenic mice with NAC resulted in a dramatic shift in the flow data pattern; isolated transgenic cells showed some staining with CD45, but a discrete leukocyte population is not evident. Similarly, low DCFH-DA staining is apparent in transgenic NAC treated samples (more so than NSC samples), but a distinct population is not evident (Fig 5). Therefore, as there is no discrete population to delineate, quadrant values were analysed. CD45+ quadrant values are significantly reduced in the NAC treated transgenic samples compared to untreated (P<0.0001) (Fig 5). The quadrant statistics for CD45+/DCFH-DA+ does not show a significant difference in the transgenic samples between NAC treatment and untreated, either in cell number or fluorescent intensity, however, absence of the discrete ROS positive leukocyte population is clearly evident from the FACS plots.

These data are consistent with the phenotypic observations and IVIS quantification of inflammation, showing that NAC treatment results in a significant reduction in the leukocyte load in the ear tissue. Furthermore, a substantial proportion of the infiltrating leukocytes in untreated transgenic mouse samples was positive for intracellular ROS and this population was reduced with NAC treatment.

### NAC acts to inhibit leukocyte recruitment to the inflamed tissue

**Fig 6:**
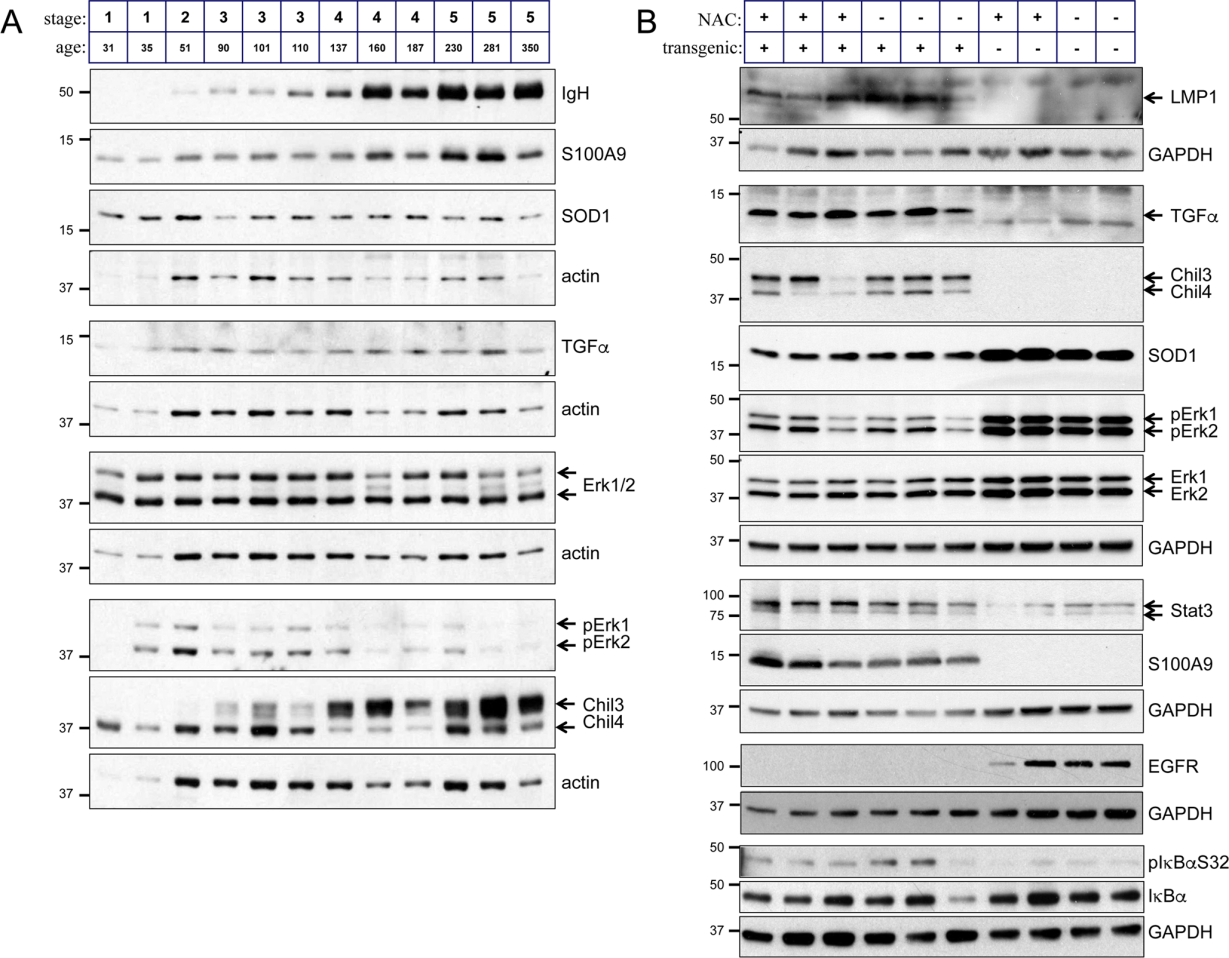
NAC treatment does not impact a set of proteins deregulated by LMP1. Ear tissue protein samples, taken from different mice were analysed by western blotting for expression of the indicated proteins. (A) Samples from L2LMP1 transgene positive mice of increasing age and stage are shown. (B) Samples were taken from NAC treated and control mice at 100 days old. LMP1 transgenic status (+ or -) and NAC treatment (+ or -) are indicated above. Actin or GAPDH was used as a loading control. Protein size markers in kD are indicated to the left of each panel.

The expression levels of numerous proteins are affected in the epithelium of the L2LMP1 transgenic mice compared to NSC and in some cases the levels are dynamic, changing as the phenotype progresses [39]. TGFα, S100A9 and Stat3 are amongst several proteins consistently induced by LMP1 in the tissue throughout the mouse life and this is not affected by NAC treatment (Fig 6). Similarly, IκBα shows increased phosphorylation (reflecting the activation of NF-κB [31]), and NAC does not affect this. Sod1, Chil3 and Chil4 (aka Chi3l3/YM1 and Chi3l4/YM2), Erk1 and 2 and EGFR (amongst others) show a dynamic expression pattern (Fig 6 and S3 Fig). Sod1, initially induced in immature transgenic samples shows lower levels compared to controls in older mice. EGFR responds to the constitutive stimulation through ligands such as TGFα, and becomes down-regulated in the transgenic inflamed tissue [31, 40]. A similar flip in Erk1 and Erk2 activation (detected by phosphorylation) is observed, over-activated in young transgenic mice, becoming down regulated in older, more advanced phenotypic stages. The inflammation-associated proteins Chil3 and Chil4, highly induced in the transgenic tissues [41], show a flip in relative abundance as the mice age. Prolonged NAC treatment (from the age of 30 days) shows no enduring impact on the expression levels of these key determinants of proliferation and inflammation (Fig 6).

**Fig 7:**
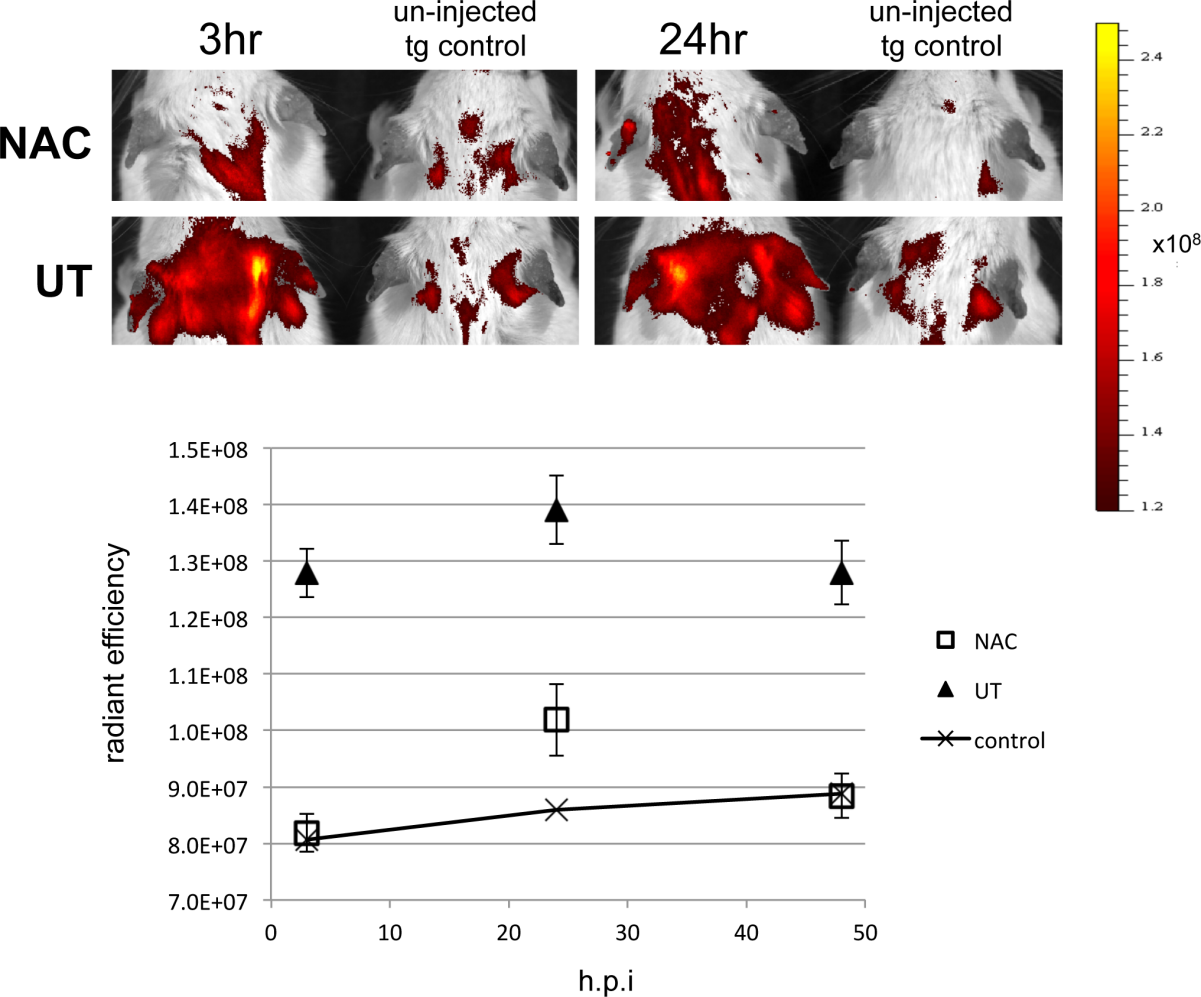
Leukocyte recruitment to the inflamed tissue correlates with phenotypic stage, which is ameliorated by NAC treatment. CPD stained leukocytes collected from inflamed L2LMP1 transgenic stage 5 ears were injected into six L2LMP1 transgenic mice (aged 103 days old), three of which had received NAC treated water (NAC) and three were not treated (UT). Mice were imaged at 3, 24 and 48 hours post injection (hpi). Top: typical epifluorescent image taken at 3 and 24 hpi. Below: the average radiant efficiency of the ROI (taken around the ear) is plotted (n = 6 for each, error bars show SEM). The average of each of the readings taken (6 repeats) for un-injected control mouse VII (Table 1), is plotted as the base line. The difference between NAC and UT is significant at each time point (p<0.0001, 0.0018, 0.0002 respectively).

It seems likely that in acting through antioxidant pathways, NAC is damping the feed forward loop between oxidative stress and inflammation. Numerous inflammatory chemokines and cytokines are upregulated in the inflamed tissue in a complex milieu, that amplifies as the phenotype progresses [30]. In order to explore the effect of NAC treatment upon leukocyte recruitment to the inflamed site, activated leukocytes [42], isolated from the ears of stage 5 L2LMP1 mice were surface stained with fluorescent dye and injected into NAC treated and untreated recipient L2LMP1 mice at approximately 100 days old (Table 1). At this age, the NAC treated mice showed a stage 2 phenotype, while untreated were at stage 3. Recruitment of the leukocytes to the inflamed site (the recipient ears) was measured by IVIS, revealing significantly higher fluorescence in the ears of transgenic untreated mice, detectable from 3 hours post injection (Fig 7 and Figure A in S3 File). Repeat of the assay in older mice of increased phenotypic stage, revealed a correlation with the ear phenotype stage and the leukocyte recruitment (Figures B and C and Table A in S3 File). These data extend the observations above, showing that NAC treatment slows the progression of the inflamed phenotype (measured by multiple parameters), and this in turn reduces leukocyte recruitment to the site, thereby tempering the escalation of the phenotype.

**Table 1:** Leukocyte recruitment. L2LMP1 transgenic mice were designated with numbers I to VII (as indicated). Treatment with NAC (from 48 days old) or untreated is indicated (+,—respectively) and ear stage at age 103 days. Mice I to VI were recipients for leukocytes (cells inj) collected from donor L2LMP1 stage 5 ears.

#	Tg	NAC	Age	stage	Cells inj
I	+	+	103	2	10^6^
II	+	+	103	2	10^6^
III	+	+	103	2	10^6^
IV	+	-	103	3	10^6^
V	+	-	103	3	10^6^
VI	+	-	103	3	10^6^
VII	+	-	103	3	none

## Discussion

EBV infection can lead to multiple post-viral inflammatory conditions as well as being a risk factor for auto-immune disorders including MS and SLE. The viral-encoded protein LMP1 is a potent inducer of inflammation [30], which may be at the root of many of these post-viral syndromes. Non-steroidal anti-inflammatory drugs are extensively used in the treatment of such conditions. Here, we have investigated the use of an anti-oxidant to boost the body’s inherent redox regulatory mechanism, in an attempt to break the feed forward loop between oxidative tissue damage and inflammation, which could persist after the virus departs.

We have used the L2LMP1 transgenic mouse model, where the skin develops a pathology of hyperplasia and increasing chronic inflammation, most severe where LMP1 is expressed to the highest level (in the skin of the ears) [28, 30, 31]. The expression levels of a large number of proteins are consequently affected, notably those involved in the control of cell proliferation and inflammatory processes [39]. As the phenotype progresses in adult mice, there is an increasing influx of immune cells and concomitant increase in the levels of multiple chemokines and cytokines released by these cells, promulgating the chronic inflammatory state [30]. We have previously shown that IgG deposition in the dermis forms part of this process and now show that it also involves the increased deposition of IgM, IgA, and IgE and activation of complement. Genetic deletion of CD40, one of the many induced factors in the pathological tissue, with accompanying loss of IgG, IgE and complement activation, reduces the characteristic degenerative features (necrosis and ulceration) of stages 4 and 5 of the phenotype. By comparison, in the complete absence of mature B and T-cells in a RAG1 null background, the phenotype was limited to stage 2 [30]. These data allow a partial separation of the role of T and B-cells in the pathology, suggesting that the B-cell factors contribute more to the tissue-destructive aspects. This is consistent with what is observed in several chronic inflammatory conditions which feature immunoglobulin deposition, such as rheumatoid arthritis, where treatments to deplete B-cells (for example Rituximab, an antibody to CD20) are effective in reducing joint damage and pain [43].

With the milieu of chemokines and cytokines involved in the inflammatory process, targeting any one may have limited effect and/or unwanted side effects. We instead sought to inhibit the feed forward loop between inflammation and oxidative stress and tissue damage, using a readily available, non-toxic antioxidant. NAC provides L-cysteine (a semi-essential amino acid) in the synthesis of the body’s natural major antioxidant, GSH, which may become depleted during inflammation. We show that long term NAC treatment reduces the leukocyte load in the inflamed tissue and thus the inflammatory pathology. This is evidenced by the visible phenotype and using a quantifiable, *in vivo* imaging assay for inflammation. The reduction in inflammation achieved through NAC treatment has the knock on effect of reducing further leukocyte recruitment to the tissue, thus damping the rate of phenotype progression.

As expected, NAC treatment did not have an impact on the expression levels of genes deregulated by LMP1 in this model, such as TGFα, S100A9 and the chitinase like proteins Chil3 and Chil4. However, any effect on signal transduction pathway components is more difficult to address. In a non-neoplastic tissue, the steady-state activation status of a pathway can be difficult to dissect, as several feedback loops are in place to counter-balance constitutive activation. For example, in TGFα overexpressing transgenic mice, with constitutive stimulation of EGFR, the levels of EGFR become down-regulated even though the pathway is still active [40]. This is also observed in the L2LMP1 transgenic mice where TGFα expression is continuously induced by LMP1 action and EGFR becomes downregulated as a consequence of persistent activation. Genetic deletion of TGFα transiently relieves the negative feedback to reveal the underlying induction of EGFR and downstream pathways [31]. If NAC treatment impacts the MAPK pathway in the inflamed tissue, it is not apparent, as EGFR down regulation, mirrored by the down regulation of Erk activation in later phenotypic stages, is not changed with NAC treatment. Similarly, NAC treatment of cultured cells has been shown to inhibit NF-κB activation through suppressing the phosphorylation of IκB leading to its degradation (an NF-κB inhibitor) [44]. With long term NAC treatment *in vivo*, we see no impact on the levels and phosphorylation of IκBα in the inflamed tissue, but as described above, as the steady-state levels reflect a balance of pathway activation and feedback regulation in the intact tissue, the underlying processes may be obscured.

## Conclusions

LMP1 is regarded as the primary oncogene of EBV in activating proliferative, cell survival and inflammatory pathways. EBV-associated cancers with expression of LMP1, notably NPC and HD, have a prominent leukocyte infiltrate. Increasing evidence suggests that a tumour leukocyte infiltrate plays a cancer cell supportive role [45]. While the use of antioxidants in cancer treatment and prevention is much debated, and the effects are likely to be cancer-type dependant, the use of NAC in the treatment of inflammation-associated and EBV/LMP1-associated cancers could be explored.

In the L2LMP1 transgenic model, the pathology is continually triggered by LMP1 expression and it is therefore not expected that any treatment (that doesn’t target LMP1 directly) would “cure” the phenotype. Nevertheless, NAC treatment slowed pathologic progression and it could prove to be a valuable treatment option for ameliorating chronic inflammatory pathologies. Indeed, with post-viral diseases and conditions, NAC treatment might facilitate resolution of the inflammation.

We have described a model of chronic inflammation of the skin, primarily displayed in the ears of the mouse. This tissue is not only readily accessible and visibly monitored, but permits quantitation of the inflammation through *in vivo* imaging. We have characterised the proteome through the progressive pathology [39], the cellular infiltrate, cytokine and chemokine profiles [30] and here, optimised an *in vivo* imaging approach. Together, these provide a comprehensive platform to assess treatment options for chronic inflammation, whatever the trigger.

## Supporting information

S1 Fig Examples of ear stage progression. The ear stage of individual L2LMP1 mice treated with NAC is compared to untreated mice.
(PDF)

S2 Fig Examination of inflamed tissue leukocytes (CD45+) for intracellular ROS. Tissue leukocytes from L2LMP1 transgenic and NSC mice, either treated with NAC or untreated, were examined for intracellular ROS using DCFH-DA and flow cytometry.
(PDF)

S3 Fig Erk1/2 and SOD1 show a dynamic expression pattern with age in the L2LMP1 transgenic inflamed tissue. Expression of Erk1/2 and Sod1 was compared between L2LMP1 and NCS young mice.
(PDF)

S1 File Optimisation of the in vivo imaging system (IVIS). In order to optimize the *in vivo* imaging system (IVIS), L2LMP1 transgenic and NSC mice were injected with 0.33nmoles to 2nmoles p680 per mouse and imaged at time points up to 48 hours post injection.
(PDF)

S2 File Quantification of the inflammatory phenotype by IVIS. The degree of inflammation was quantified by IVIS in L2LMP1 transgenic and NSC mice, in four age groups, with and without NAC treatment. Strategies to measure the radiant efficiency were compared and the freehand drawn circumference of the region of interest (ROI) was selected as the most appropriate measure.
(PDF)

S3 File NAC treatment reduces leukocyte recruitment to the site of inflammation in L2 LMP1 mice. CPD stained leukocytes were injected into L2LMP1 transgenic and NSC mice, either treated with NAC or untreated, and their passage to the inflamed site was followed by IVIS.
(PDF)
