# Supplementary material for: N-acetylcysteine (NAC) ameliorates Epstein-Barr virus latent membrane protein 1 induced chronic inflammation
Source: PLoS One. 2017 Dec 11;12(12):e0189167. doi: 10.1371/journal.pone.0189167 (PMC5724866; doi:10.1371/journal.pone.0189167)
Supplement: S1 Fig — The ear stage of individual L2LMP1 mice treated with NAC is compared to untreated mice. (PDF) [file pone.0189167.s001.pdf]

S1 Fig

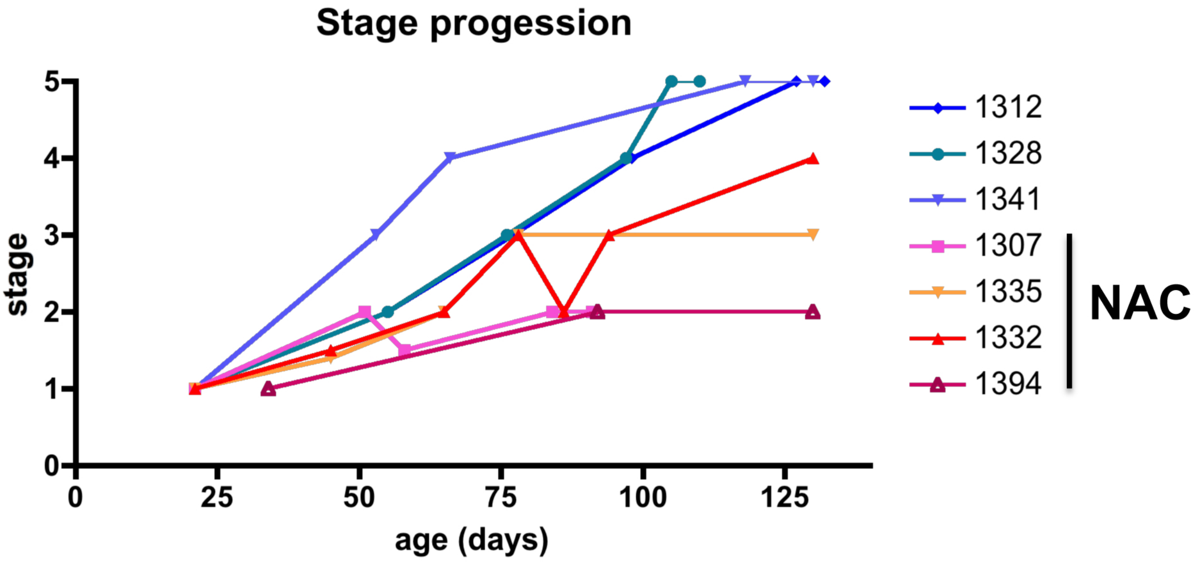

S1 Fig Examples of ear stage progression

Ear stage progression is shown for three untreated L2LMP1 transgenic mice (ID: 1312, 1328,, 1341) and four NAC treated L2LMP1 transgenic mice (ID 1307, 1335, 1332, 1394). Mouse 1394 was in a cage treated from birth and did not progress beyond stage 2 in the study period (which was also observed in other mice treated from birth). Mice 1307, 1335 and 1332 were NAC treated from 1 month old. Note: phenotype stage reversal for both 1307 and 1332, followed by stage progression.
