# Supplementary material for: N-acetylcysteine (NAC) ameliorates Epstein-Barr virus latent membrane protein 1 induced chronic inflammation
Source: PLoS One. 2017 Dec 11;12(12):e0189167. doi: 10.1371/journal.pone.0189167 (PMC5724866; doi:10.1371/journal.pone.0189167)
Supplement: S2 Fig — Tissue leukocytes from L2LMP1 transgenic and NSC mice, either treated with NAC or untreated, were examined for intracellular ROS using DCFH-DA and flow cytometry. (PDF) [file pone.0189167.s002.pdf]

S2 Fig Examination of inflamed tissue leukocytes (CD45+) for intracellular ROS

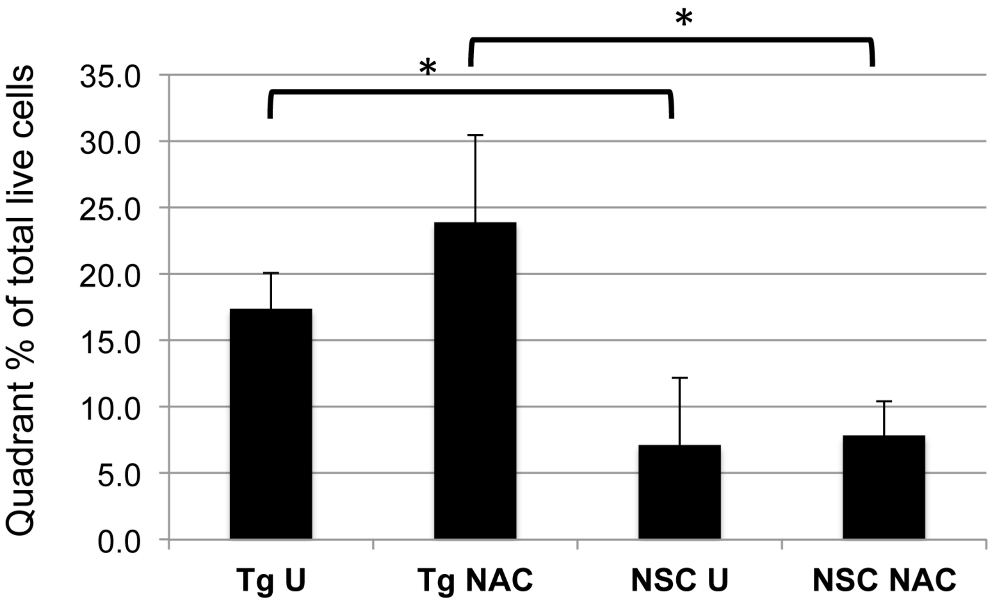

age	Tg untreated		Tg NAC		NSC untreated	NSC NAC
	n	stage	n	stage	n	n
105	7	3/4	6	2	3	3

S2 Figure CD45+/DCFH-DA+ quadrant statistics

L2LMP1 transgenic (Tg) and transgene-negative sibling controls (NSC) mice were either untreated (U) or treated with NAC in the drinking water from 1 month of age. The number of mice under study (n) and phenotypic stage of the ears are indicated in the table below the graph. Cells were isolated from the whole ear tissues and analysed by flow cytometry. Dead cells staining positive for 7AAD were gated out. The proportion of cells falling in the CD45+ (leukocytes) and DCFH-DA+ (intracellular ROS) double positive quadrant have been graphed. However, it should be noted that only untreated transgenic positive samples (Tg U) showed a discrete population of CD45+/DCFH-DA+ cells. Error bars show standard deviation.

* Transgenic sample values are significantly different from NSC (Tg U : NSC U, P=0.0026; Tg NAC: NSC NAC, P=0.0054
