# Supplementary material for: N-acetylcysteine (NAC) ameliorates Epstein-Barr virus latent membrane protein 1 induced chronic inflammation
Source: PLoS One. 2017 Dec 11;12(12):e0189167. doi: 10.1371/journal.pone.0189167 (PMC5724866; doi:10.1371/journal.pone.0189167)
Supplement: S3 Fig — Expression of Erk1/2 and Sod1 was compared between L2LMP1 and NCS young mice. (PDF) [file pone.0189167.s003.pdf]

S3 Fig. Erk1/2 and Sod1 show a dynamic expression pattern with age in the L2LMP1 transgenic inflamed tissue

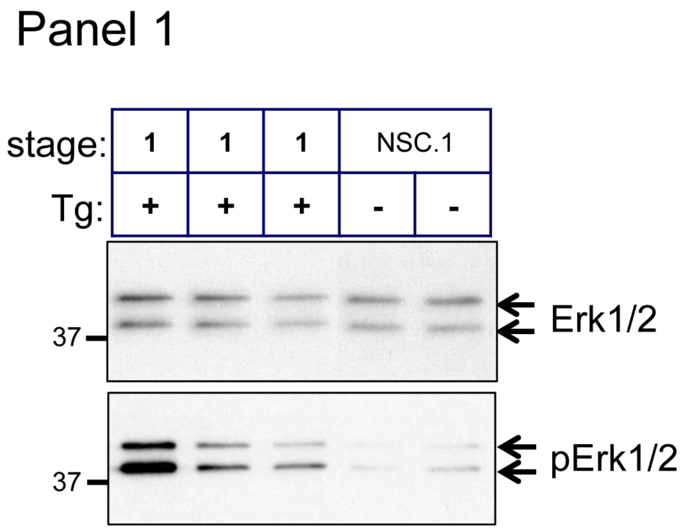

Panel 1: In young mice (45 days old) when the transgenic (Tg: +) L2LMP1 show an ear stage 1 phenotype Erk1 and 2 show greater activation (phosphorylation) in the transgenic samples compared to controls. With age, EGFR becomes downregulated (Charalambous *et al*, (2007), Carcinogenesis 28:1839), as a result of the persistent stimulation through ligands including TGFα, and thence negative feedback control. As a consequence, the activation of Erk1/2 (by phosphorylation), becomes down regulated compared to controls at later phenotypic stages (see Fig 6). Protein size markers in kD are indicated to the left of each panel.

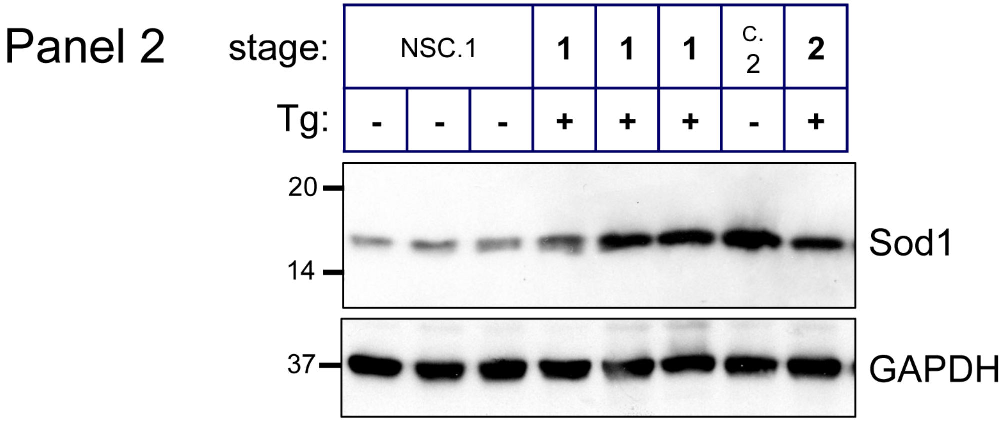

Panel 2: From a proteomic comparison (Hannigan *et al*, (2007) J. Proteome research, 6:3422), Sod1 was found to be significantly down-regulated in the L2LMP1 transgenic, inflamed tissue at stage 5 compared to age-matched transgene-negative sibling controls (see Fig 6). However, in young transgenic mice (Tg: +) at stage 1 (45 days old), Sod1 is elevated compared to age-matched transgene-negative sibling controls (NSC.1). The reversal of Sod1 levels (from upregulated to down regulated), begins at early stage 2. Protein size markers in kD are indicated to the left of each panel.
