# Supplementary material for: N-acetylcysteine (NAC) ameliorates Epstein-Barr virus latent membrane protein 1 induced chronic inflammation
Source: PLoS One. 2017 Dec 11;12(12):e0189167. doi: 10.1371/journal.pone.0189167 (PMC5724866; doi:10.1371/journal.pone.0189167)

S1 File: Supplementary information file 1
Optimisation of the *in vivo* imaging system (IVIS)

In order to optimize the *in vivo* imaging system (IVIS), L2LMP1 transgenic and NSC mice were injected with 0.33nmoles to 2nmoles p680 per mouse and imaged at time points up to 48 hours post injection (hpi). Increasing the quantity of p680 used from 0.33nmoles to 0.66nmoles/mouse resulted in a substantial increase in fluorescent signal at all time points examined, however, the increase in signal was less apparent (or not at all) using greater than 0.66nmoles p680. Subsequently an optimal range of 0.6 to 1nmole/mouse was chosen. At this quantity, the fluorescent signal from the ears peaked between 24 and 36 hpi.

Figure A
An L2LMP1 transgenic (tg) mouse at stage 3 (St3) and transgenic-negative sibling control (NSC) were injected intravenously with 2nmoles of p680. *In vivo* imaging was performed at 12, 24, 36 and 48 hpi (as indicated). Panel 1: The data are shown as line graphs where each data point represents the mean fluorescent signal emitted per ear (measured as the ratio of light emitted (photons/sec/area) to incident light (photons/sec/area) or ((photons/sec/cm^2/sr)/(µW/cm^2)). Note: the x-axis, radiant efficiency is shown on logarithmic scale. Panel 2: Images showing the fluorescent signal emitted from the skin of the head, most notably the ears, for NSC and stage 3 transgenic.

Figure B
Four L2LMP1 transgenic (tg) mice at stage 2 (St2) phenotype were injected intravenously with either 0.33, 0.66, 0.99 or 1.33nmoles of p680 (as indicated). *In vivo* imaging was performed from 3 to 48 hpi (as indicated).

Figure C
Four L2LMP1 transgenic (tg) mice at stage 4 (St4) phenotype were injected intravenously with either 0.33, 0.66, 0.99 or 1.33nmoles of p680 (as indicated). *In vivo* imaging was performed from 3 to 48 hpi (as indicated).

Figure D
Fluorescent signals from the ears (mean value of the two from each mouse) from the eight L2LMP1 transgenic (tg) mice at stage 2 or 4 phenotype (Panels 1 and 2 respectively), images shown in Figures B and C, have been quantified and shown as line graphs.

Figure A

Panel 1

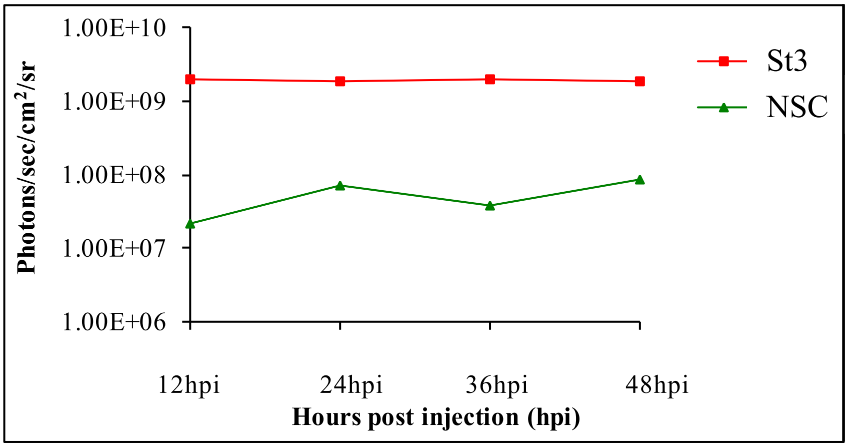

Panel 2

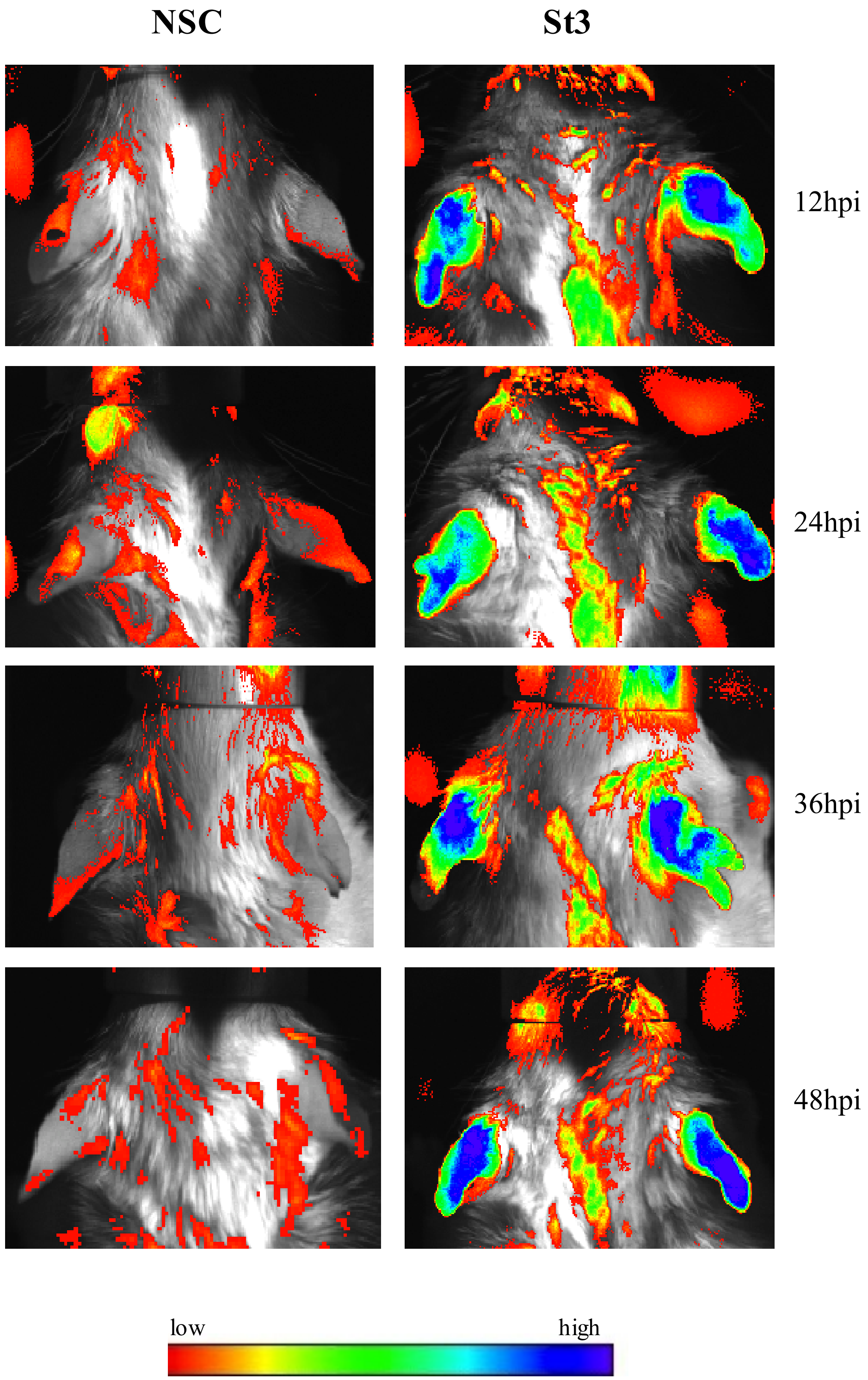

Figure B
St2

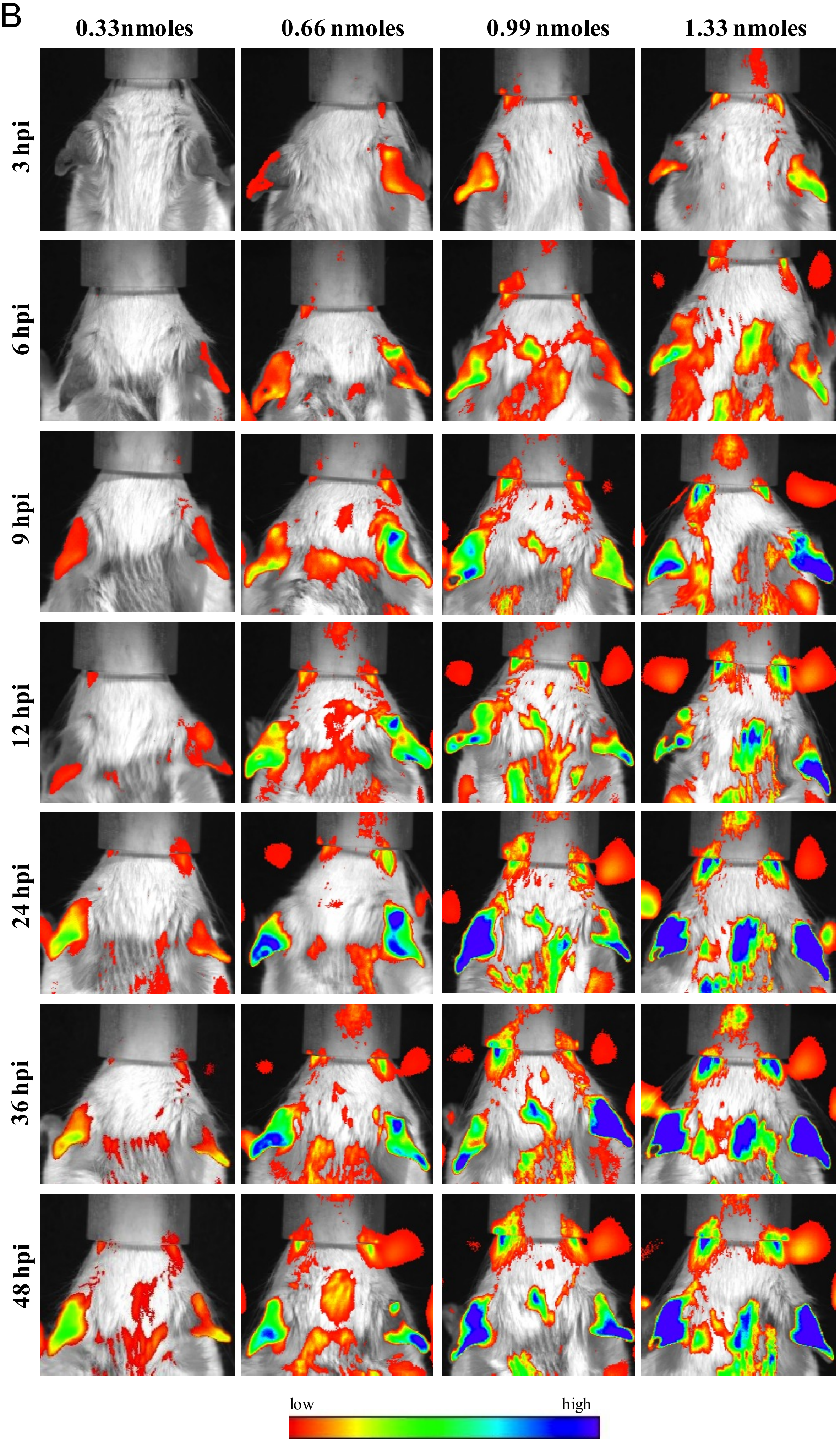
0.33nmoles
0.66 nmoles
0.99 nmoles
1.33 nmoles
3 hpi
6 hpi
9 hpi
12 hpi
24 hpi
36 hpi
48 hpi
low
high

Figure C
St4

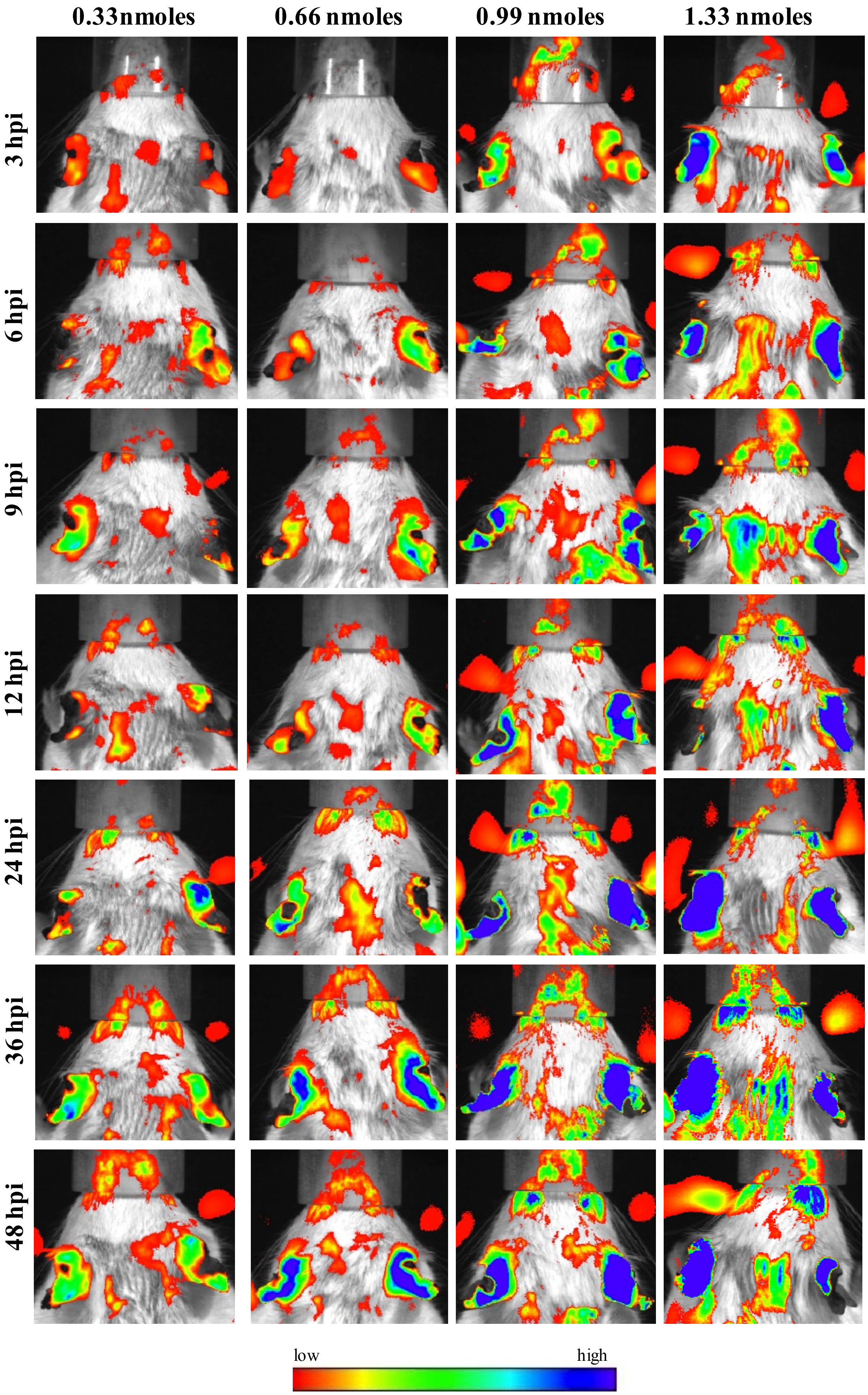
0.33nmoles
0.66 nmoles
0.99 nmoles
1.33 nmoles
3 hpi
6 hpi
9 hpi
12 hpi
24 hpi
36 hpi
48 hpi
low
high

Figure D

Panel 1

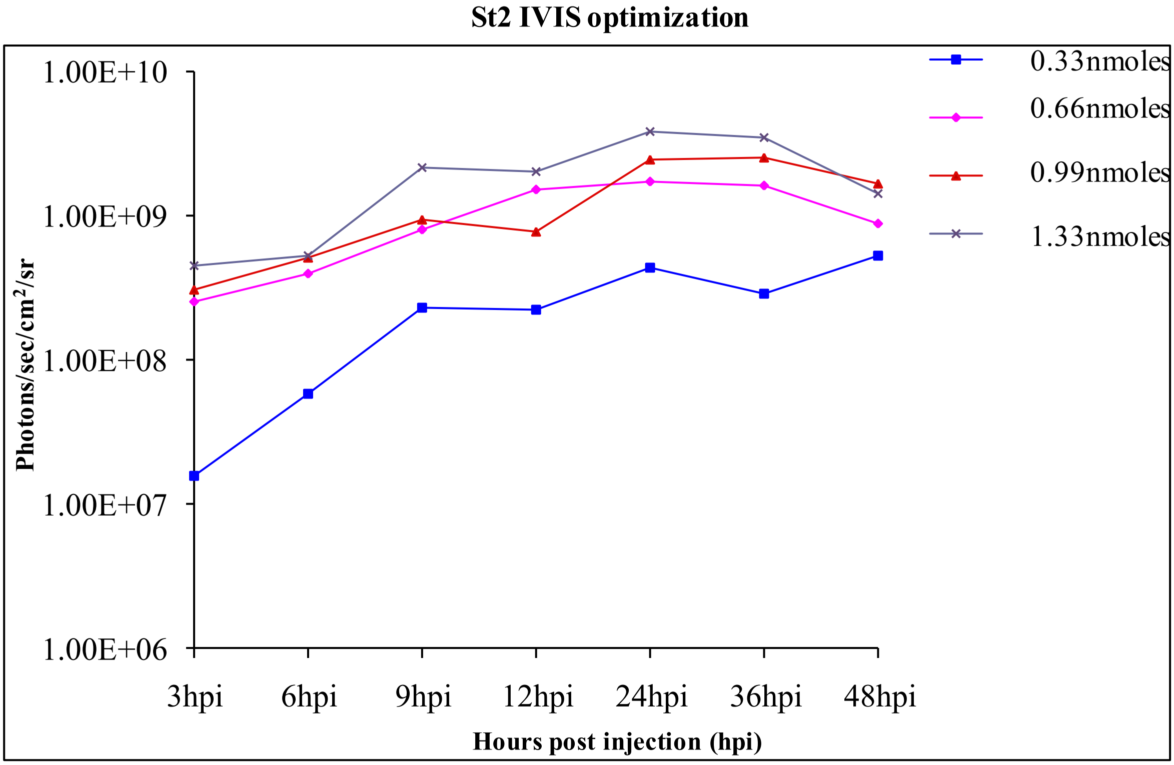

Panel 2

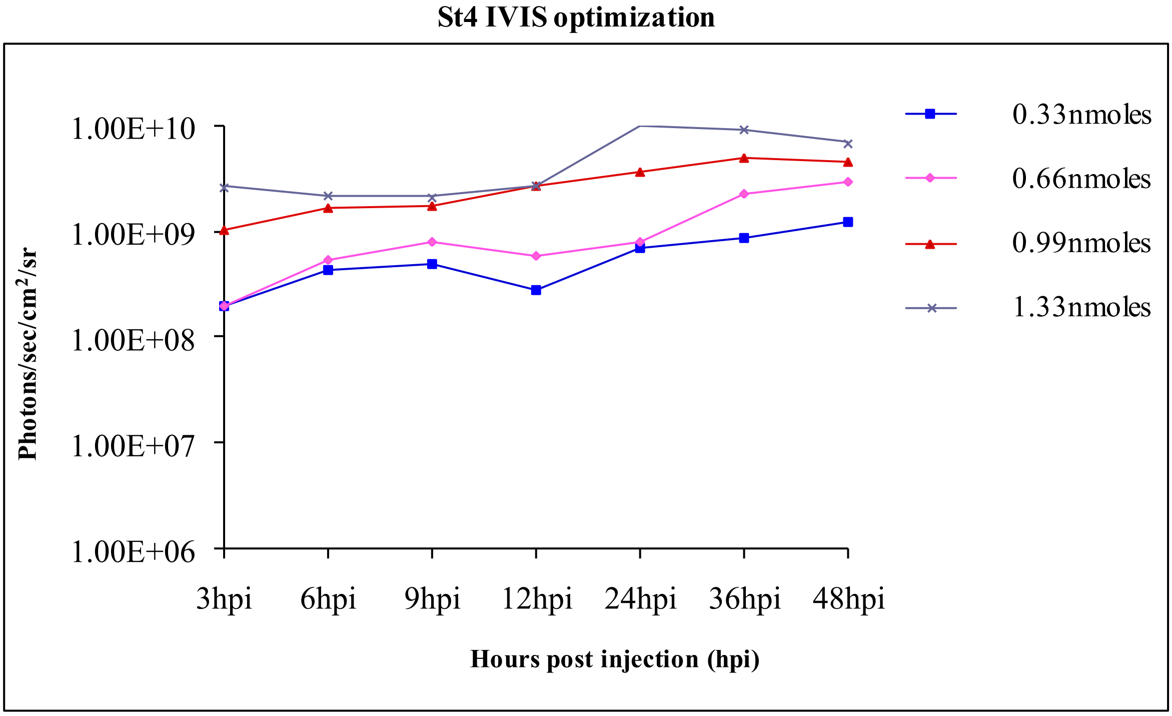

Supplement: S1 File — In order to optimize the in vivo imaging system (IVIS), L2LMP1 transgenic and NSC mice were injected with 0.33nmoles to 2nmoles p680 per mouse and imaged at time points up to 48 hours post injection. (PDF) [file pone.0189167.s004.pdf]
