# Supplementary material for: N-acetylcysteine (NAC) ameliorates Epstein-Barr virus latent membrane protein 1 induced chronic inflammation
Source: PLoS One. 2017 Dec 11;12(12):e0189167. doi: 10.1371/journal.pone.0189167 (PMC5724866; doi:10.1371/journal.pone.0189167)
Supplement: S2 File — The degree of inflammation was quantified by IVIS in L2LMP1 transgenic and NSC mice, in four age groups, with and without NAC treatment. Strategies to measure the radiant efficiency were compared and the freehand drawn circumference of the region of interest (ROI) was selected as the most appropriate measure. (PDF) [file pone.0189167.s005.pdf]

S2 File: Supplementary information file 2
Quantification of the inflammatory phenotype by IVIS

In the following series of figures (Figures A to D), example mice from each of the age groups (57, 80, 100 and 130 days old) and in each of the four test categories: L2LMP1 transgenic (Tg) and control (NSC), either treated with NAC in the drinking water from 1 month old (N), or untreated (U) are shown.

In each case, the mice were injected with 1 nmol p680 and then imaged *in vivo* at intervals post injection: 3, 6, 9, 12, 18, 24, 30 and 36 hours.

The number of ears imaged (n, ie the number of mice x 2) and phenotypic stage in each group are shown in the table below (table A).

In the images shown, the region of interest (ROI) is depicted as a “freehand drawn” circumference around each ear (blue line). The radiant efficiency is measured in (photons/sec/cm^2/sr)/(µW/cm^2).

Table A Age, phenotypic stage and numbers imaged in this experiment

age	Tg untreated		Tg NAC		NSC untreated	NSC NAC
	n	stage	n	stage	n	n
57	4	1	4	1	4	4
80	2	2	2	1.5	2	2
100	2	3.5	2	2	2	2
130	6	4.5	8	2.5	6	

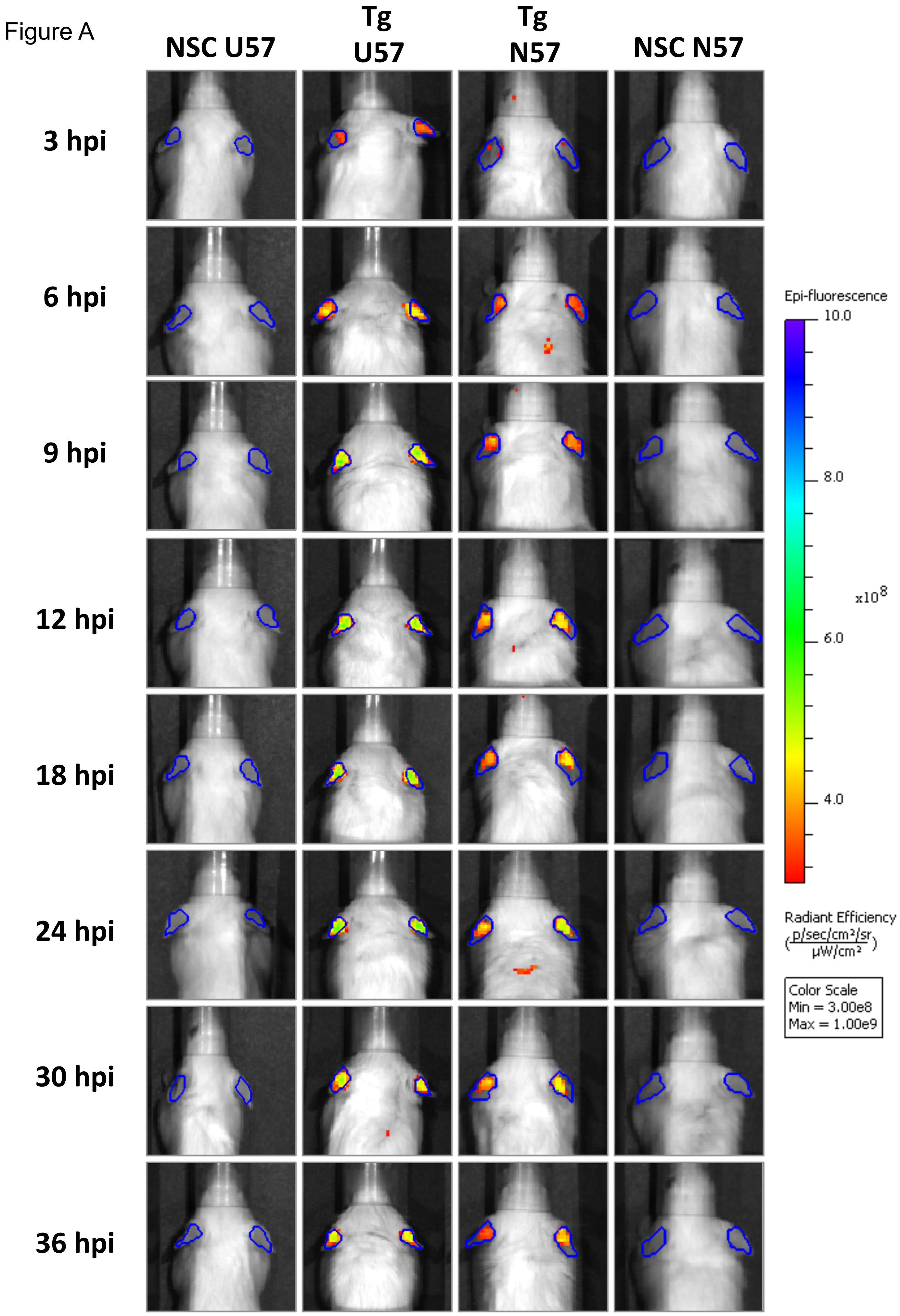

Figure A
NSC U57
Tg U57
Tg N57
NSC N57
3 hpi
6 hpi
9 hpi
12 hpi
18 hpi
24 hpi
30 hpi
36 hpi
Epi-fluorescence
10.0
8.0
6.0
4.0
x10^8
Radiant Efficiency
(p/sec/cm²/sr / µW/cm²)
Color Scale
Min = 3.00e8
Max = 1.00e9

Figure B

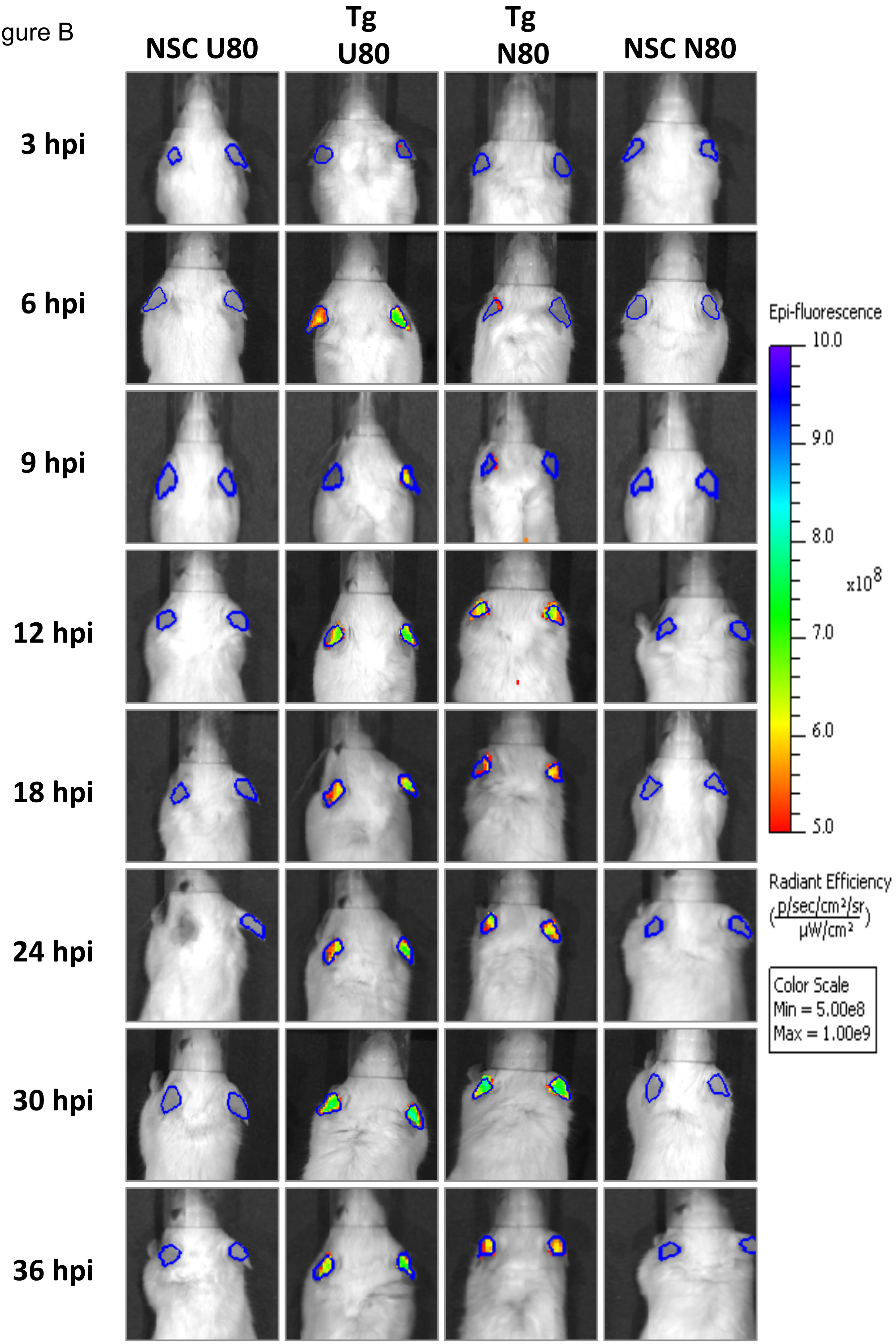
NSC U80
Tg U80
Tg N80
NSC N80
3 hpi
6 hpi
9 hpi
12 hpi
18 hpi
24 hpi
30 hpi
36 hpi
Epi-fluorescence
10.0
9.0
8.0
x10^8
7.0
6.0
5.0
Radiant Efficiency
(p/sec/cm²/sr / μW/cm²)
Color Scale
Min = 5.00e8
Max = 1.00e9

Figure C

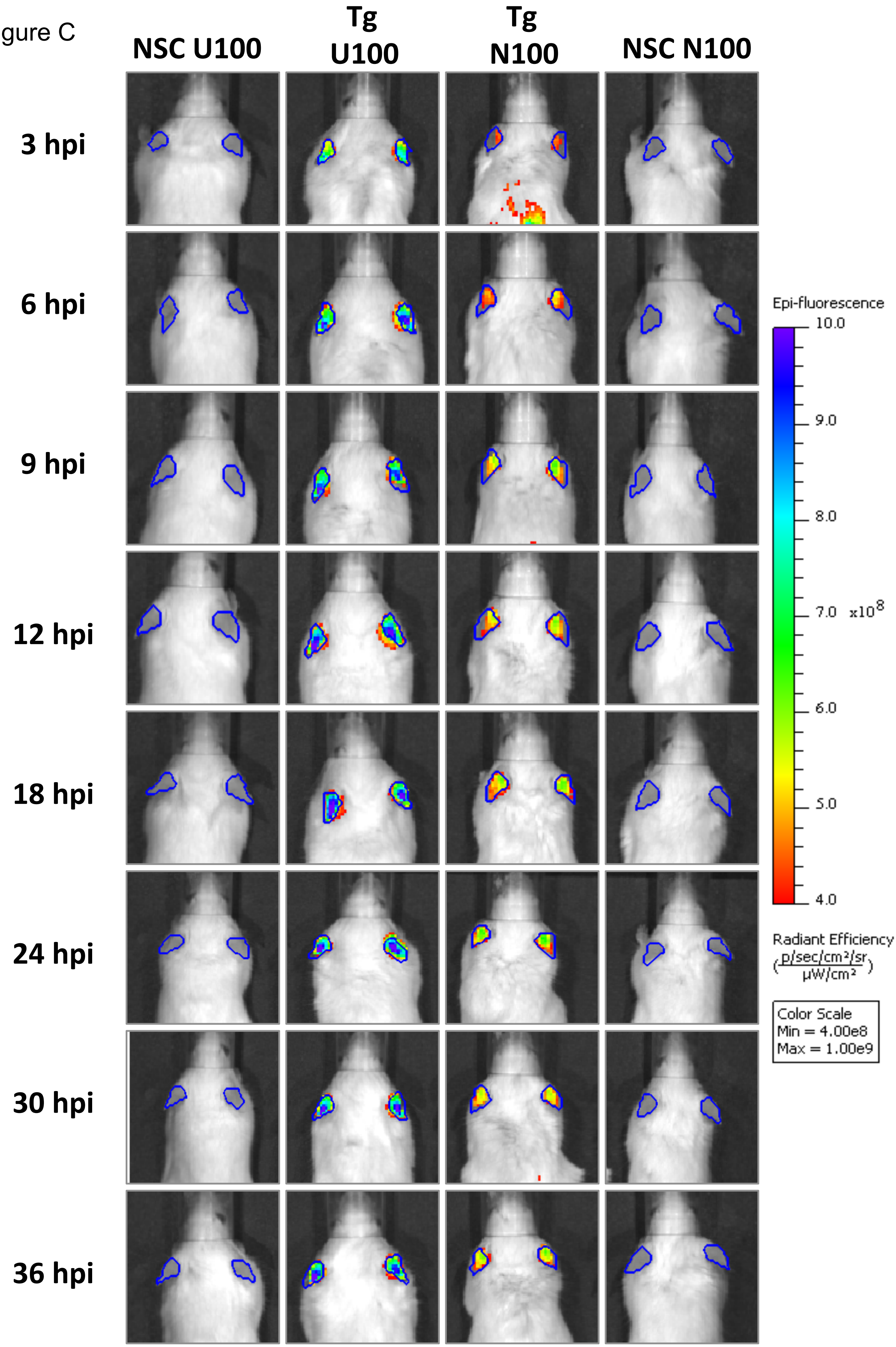
NSC U100
Tg U100
Tg N100
NSC N100
3 hpi
6 hpi
9 hpi
12 hpi
18 hpi
24 hpi
30 hpi
36 hpi
Epi-fluorescence
10.0
9.0
8.0
7.0 x10^8
6.0
5.0
4.0
Radiant Efficiency
(p/sec/cm²/sr / μW/cm²)
Color Scale
Min = 4.00e8
Max = 1.00e9

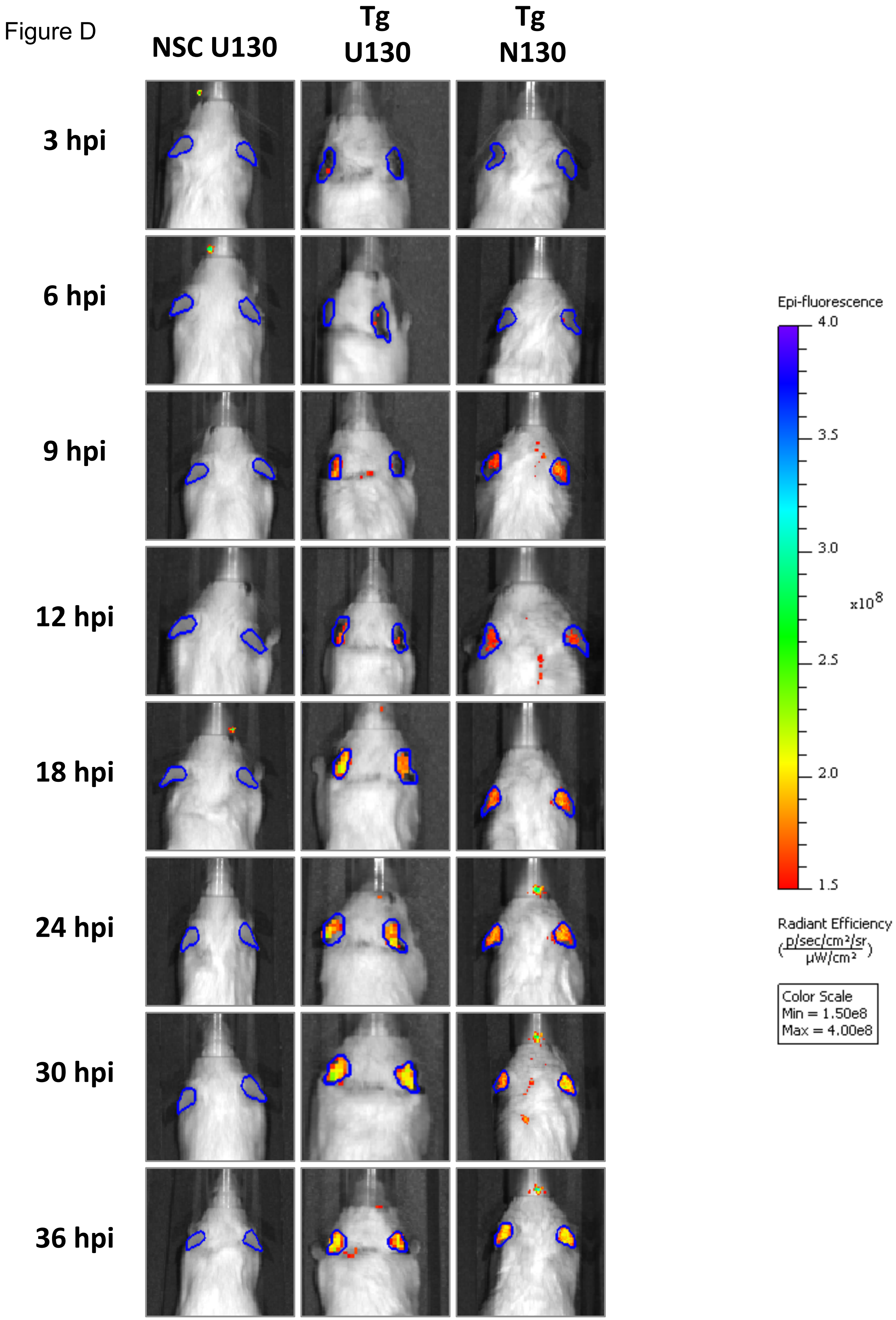

Figure D
NSC U130
Tg U130
Tg N130
3 hpi
6 hpi
9 hpi
12 hpi
18 hpi
24 hpi
30 hpi
36 hpi
Epi-fluorescence
4.0
3.5
3.0
2.5
2.0
1.5
x10^8
Radiant Efficiency
(p/sec/cm²/sr / µW/cm²)
Color Scale
Min = 1.50e8
Max = 4.00e8

Figure E

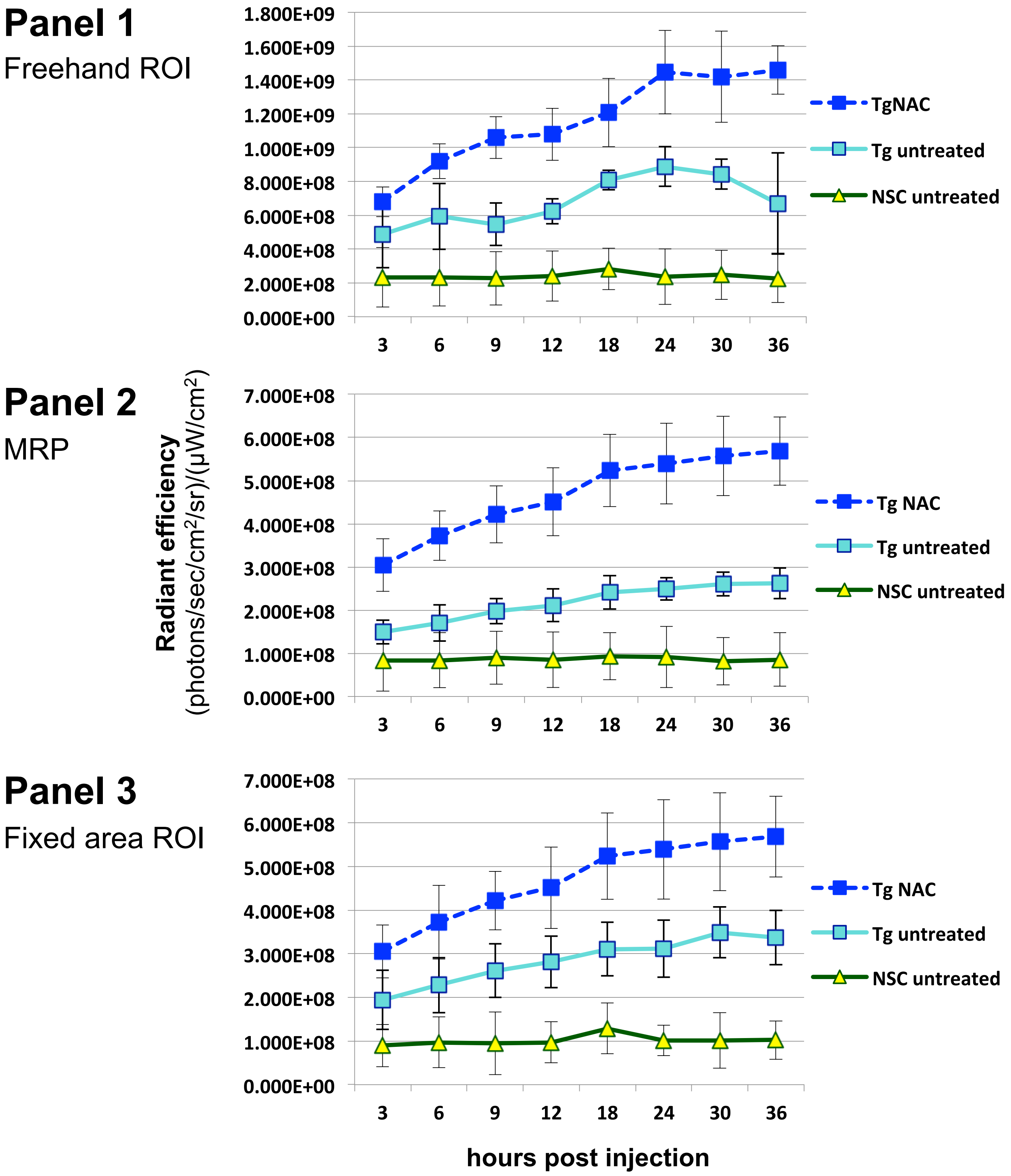

An appropriate strategy to measure and compare the fluorescent signal (radiant efficiency: (photons/ sec/cm^2/sr)/(µW/cm2) from the ears was explored. Transgenic mice treated with NAC (4 mice, 8 ears), transgenic mice untreated (3 mice, 6 ears) and NSC untreated mice (3 mice, 6 ears) aged 130 days old were injected with p680 and imaged at intervals from 3 to 36 hours post injection (hpi) as indicated. Three alternative analytical approaches were examined. Panel 1: defining the region of interest (ROI) as the relative signal derived from the whole ear (as delineated by freehand drawn circumference). Panel 2: the maximum radiance pixel (MRP) from within the ROI. Panel 3: the ROI signal derived from a fixed circular area, smaller than the full ear area and centred on the MRP. All three measurements gave data showing the same trend. Therefore, in order to reflect the inflamed state of the ear tissue as a whole, the approach shown in Panel 1 (freehand drawn circumference) was used in further analyses.
Error bars show SD.

Figure F

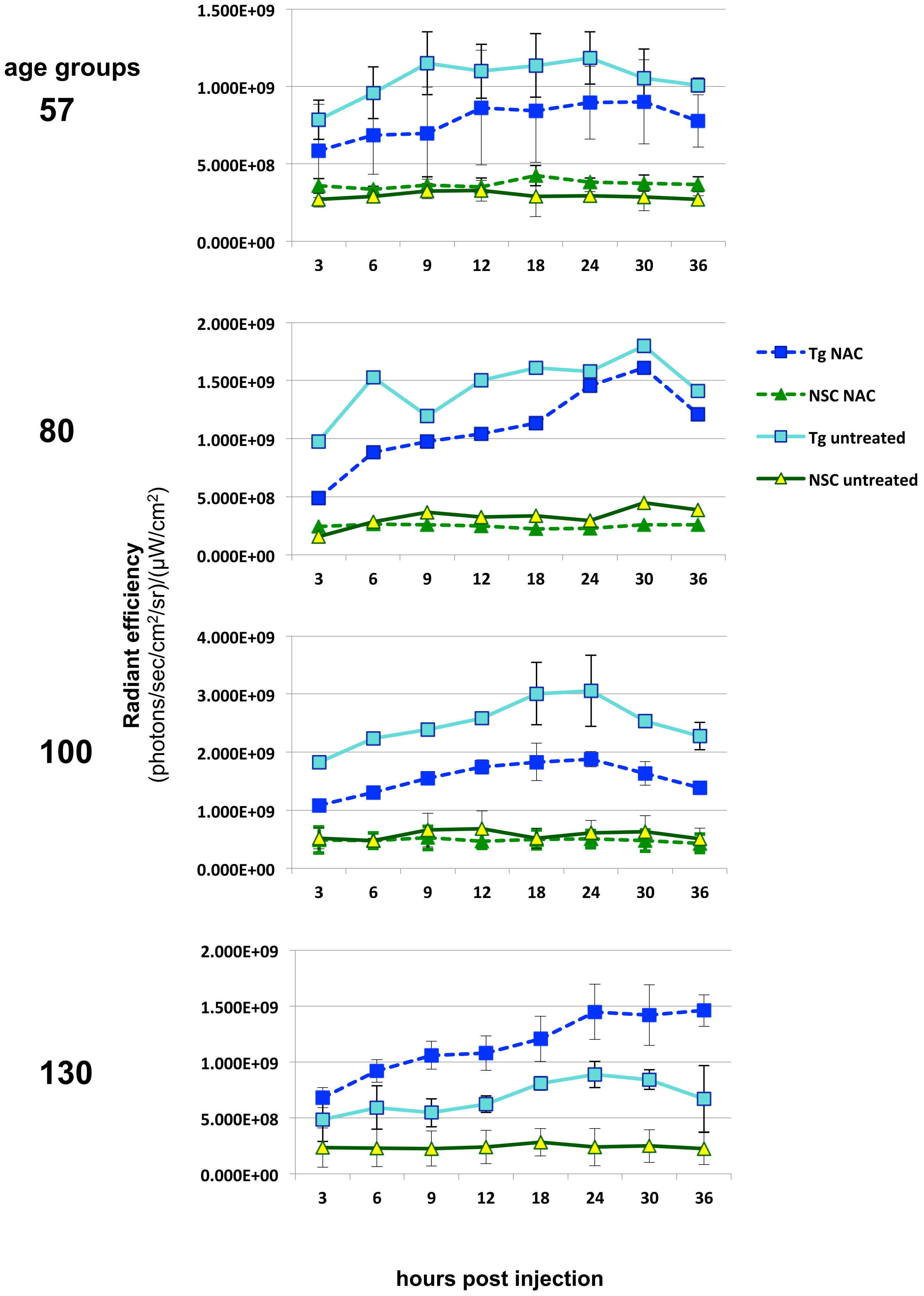
age groups
57
80
100
130
Radiant efficiency
(photons/sec/cm2/sr)/(µW/cm2)
hours post injection
Tg NAC
NSC NAC
Tg untreated
NSC untreated
1.500E+09
1.000E+09
5.000E+08
0.000E+00
2.000E+09
4.000E+09
3.000E+09
3
6
9
12
18
24
30
36

Figure G

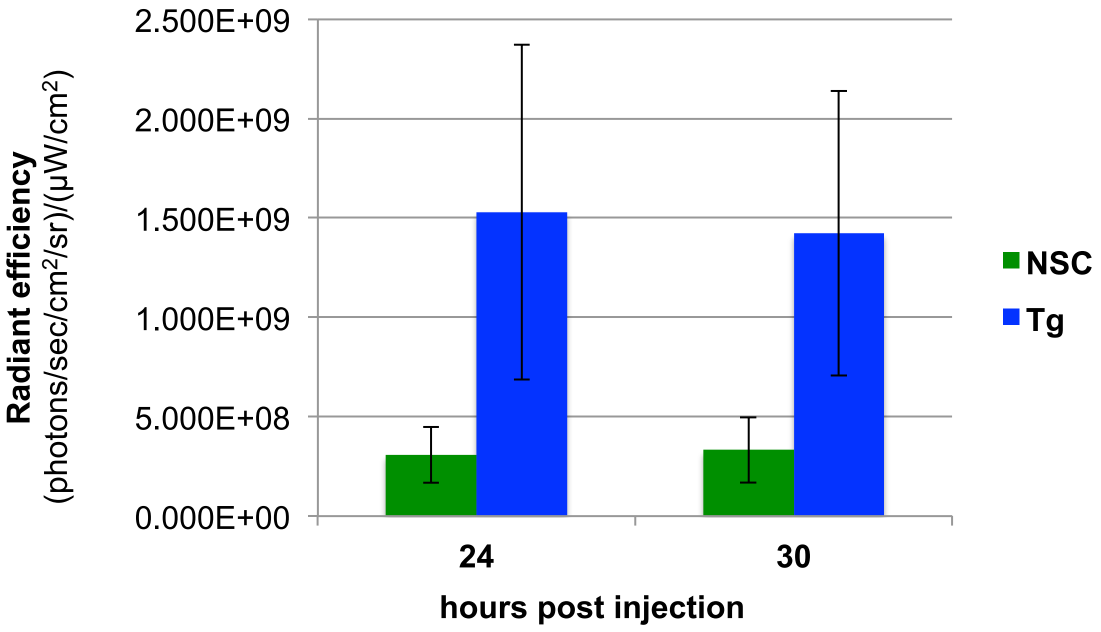

Figure F
L2LMP1 transgenic mice (Tg) and NSC mice, treated with NAC or untreated were assessed by IVIS following p680 injection. Mice at 57, 80, 100 and 130 days old (as indicated) were analysed (see Table A). Images were taken at intervals between 3 and 36 hpi as indicated. The mean radiant efficiency of the region of interest (ROI) is plotted using the freehand drawn circumference (shown in Panel 1 in Figure E). Error bars indicate SD. Treatment with NAC reduces the transgenic signal in mice up to approximately 100 days of age. Beyond this age, the signal from untreated mice reduces as the tissue becomes necrotic and eroded, while the NAC treated mice lag behind in the progression of the phenotype.

Figure G
L2LMP1 transgenic mice show an order of magnitude higher p680 fluorescence in the ears compared to NSC, at all ages ($p<0.0001$ at both 24 and 30 hpi). Data from untreated transgenic (Tg, n=16) and NSC (n=16) mice of all ages are compared at 24 and 30 hpi. Error bars indicate SD. The higher SD observed in transgenic samples is due to the noted increasing signal from age 57 to 100 days, followed by a declining signal as the mice age further, while the NSC signal remains relatively constant across all ages.
