# Supplementary material for: N-acetylcysteine (NAC) ameliorates Epstein-Barr virus latent membrane protein 1 induced chronic inflammation
Source: PLoS One. 2017 Dec 11;12(12):e0189167. doi: 10.1371/journal.pone.0189167 (PMC5724866; doi:10.1371/journal.pone.0189167)

S3 File: Supplementary information file 3
NAC treatment reduces leukocyte recruitment to the site of inflammation in L2LMP1 mice

Figure A

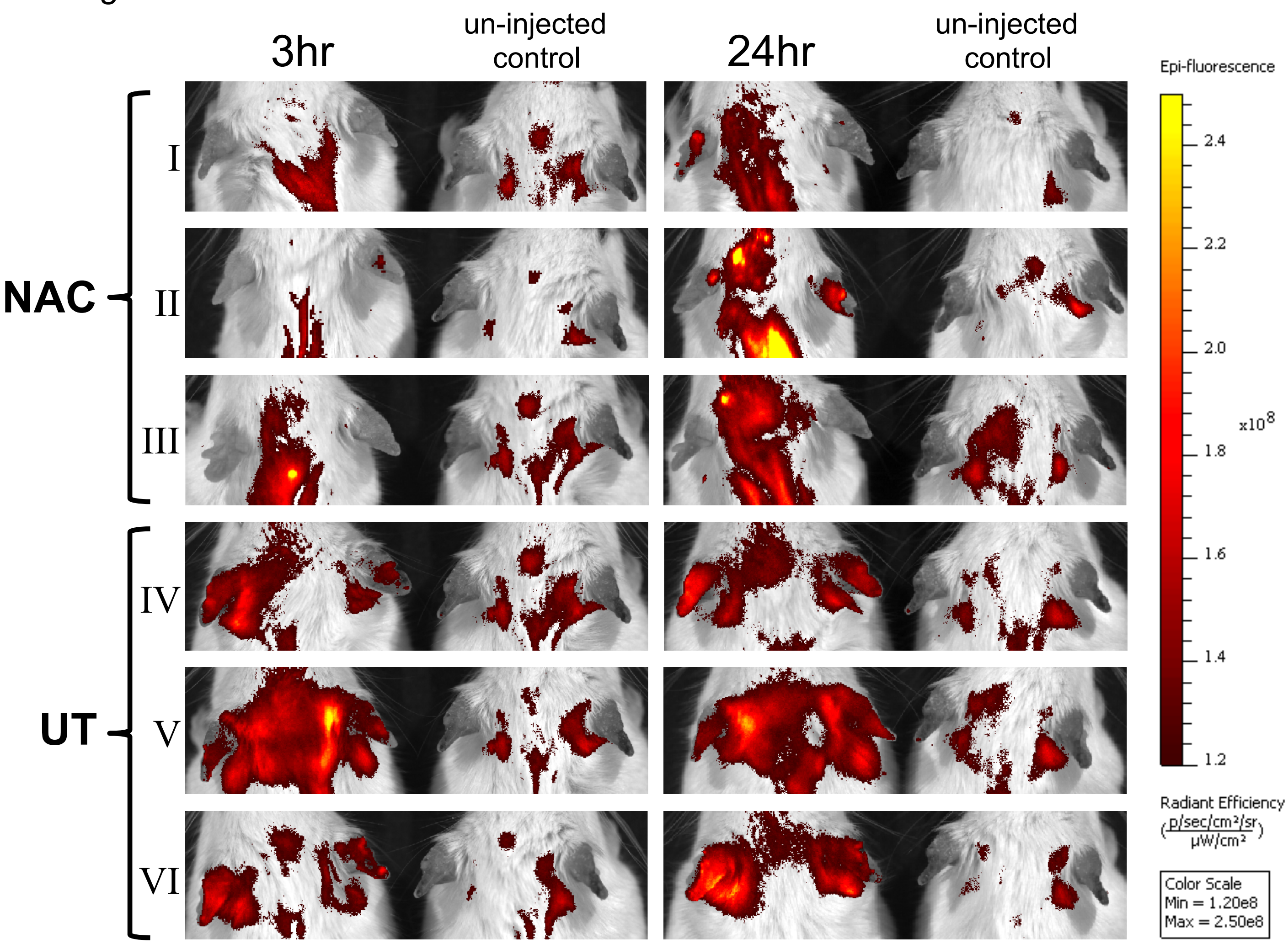

Figure A. NAC treatment reduces leukocyte recruitment to the site of inflammation.
10^6 CPD stained leukocytes collected from inflamed L2LMP1 transgenic stage 5 ears were injected IV (tail vein) into six L2LMP1 transgenic mice (aged 103 days old), three of which (I, II, III) had received NAC treated water from 48 days old (upper panels, left image in each case) and three (IV, V, VI) were not treated (UT: lower panels, left image in each case). Mice were imaged at 3, 24 and 48 hours post injection (3 and 24 shown). In each image panel, the same control, L2LMP1 transgenic mouse not injected with cells is shown to the right (un-injected control, VII in Table A), showing the level of auto-fluorescence. Note: by 3 hours, the labeled cells have reached the inflamed skin of the head and ears in the untreated transgenic mice, but are not yet evident in the ears of NAC treated transgenic mice. By 24 hours, the fluorescent leukocytes have just begun to reach the ears in the NAC treated mice.

Table A and Figures B and C. Leukocyte recruitment to the site of inflammation correlates with the extant degree of inflammation.

In the cohort of mice (as detailed in Table A), 10^6 CPD stained leukocytes collected from inflamed L2LMP1 transgenic stage 5 ears were injected IV (tail vein) into six L2LMP1 transgenic mice (aged 131 days old), four of which had received NAC treated water from 48 days old (NAC) and two were not treated (UT). Mice were imaged at 1, 3, 6, 9 and 24 hours post injection (hpi) and the average radiant efficiency of the ROI (taken around the ear) is plotted in Figure B (ears n=8 and n=4 respectively, error bars show SEM). The average of each of the readings taken (6 repeats) for uninjected control mouse X, is plotted as the base line. The difference between NAC and UT is significant at 1 and 3 hpi ($p<0.016$, 0.042, 0.23, 0.095, 0.59 for each time point respectively). Figure C: Example images showing phenotype stage and fluorescence at 24 hpi of three NAC treated mice (for ear stage, see Table A). Note, leukocyte recruitment to the ear, correlates with the phenotypic stage, including when the stage of the two ears differ within the same mouse, as in mouse II.

Table A

#	Tg	NAC	Age inj-1st	stage	Age inj-2nd	stage
I	+	+	103	2		
II	+	+	103	2	131	2 | 3.5
III	+	+	103	2	131	2
IV	+	-	103	3		
V	+	-	103	3	131	3
VI	+	-	103	3		
VII	+	-	control	3	131	3.5
VIII	+	+		2	131	3.5 | 4
IX	+	+		2	131	2
X	-	-		-	control	-

Figure C

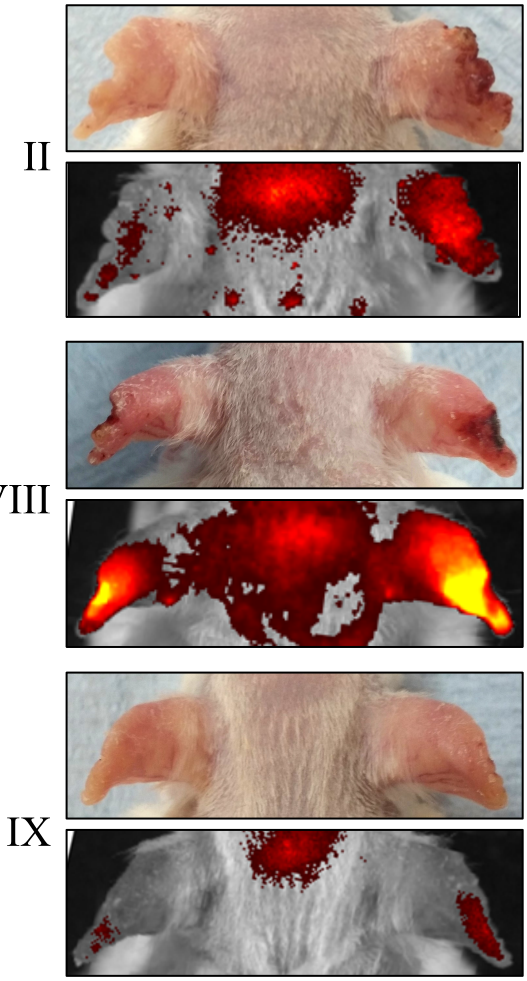

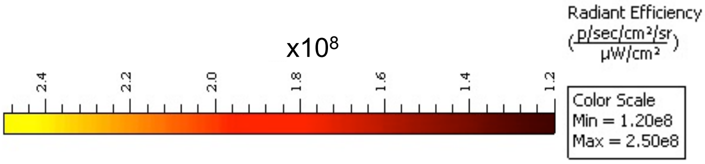

Figure B

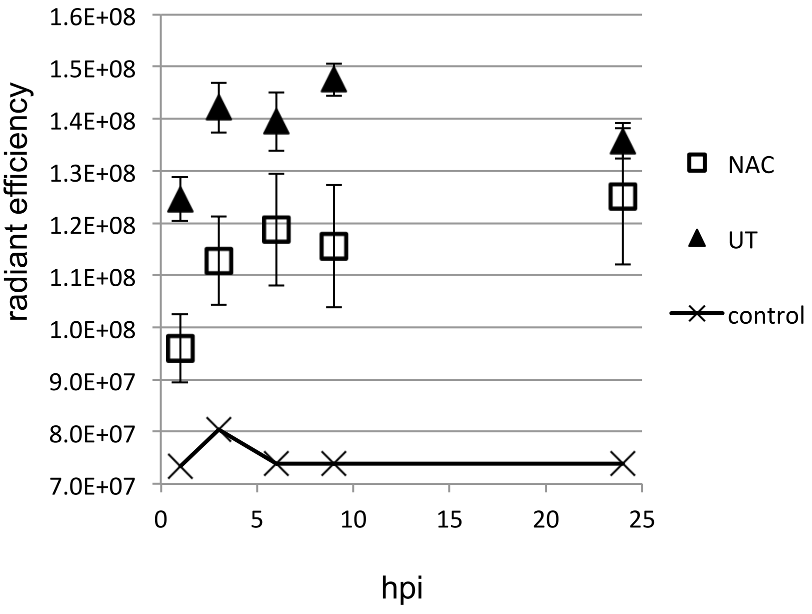

Supplement: S3 File — CPD stained leukocytes were injected into L2LMP1 transgenic and NSC mice, either treated with NAC or untreated, and their passage to the inflamed site was followed by IVIS. (PDF) [file pone.0189167.s006.pdf]
